# Altered Hub Functioning and Compensatory Activations in the Connectome: A Meta-Analysis of Functional Neuroimaging Studies in Schizophrenia

**DOI:** 10.1093/schbul/sbv146

**Published:** 2015-10-15

**Authors:** Nicolas A. Crossley, Andrea Mechelli, Cedric Ginestet, Mikail Rubinov, Edward T. Bullmore, Philip McGuire

**Affiliations:** ^1^Department of Psychosis Studies, Institute of Psychiatry, Psychology and Neuroscience, King’s College London, London, UK;; ^2^Department of Neuroimaging Studies, Institute of Psychiatry, Psychology and Neuroscience, King’s College London, London, UK;; ^3^Department of Mathematics and Statistics, Boston University, Boston, MA;; ^4^Behavioural and Clinical Neuroscience Institute, Department of Psychiatry, University of Cambridge, Cambridge, UK;; ^5^Cambridgeshire and Peterborough NHS Foundation Trust, Cambridge, UK;; ^6^ImmunoPsychiatry, Alternative Discovery and Development, GlaxoSmithKline, Cambridge, UK; ^7^These authors contributed equally to this work.

**Keywords:** schizophrenia, connectome, fMRI, graph analysis, hubs

## Abstract

**Background:**

Functional neuroimaging studies of schizophrenia have identified abnormal activations in many brain regions. In an effort to interpret these findings from a network perspective, we carried out a meta-analysis of this literature, mapping anatomical locations of under- and over-activation to the topology of a normative human functional connectome.

**Methods:**

We included 314 task-based functional neuroimaging studies including more than 5000 patients with schizophrenia and over 5000 controls. Coordinates of significant under- or over-activations in patients relative to controls were mapped to nodes of a normative connectome defined by a prior meta-analysis of 1641 functional neuroimaging studies of task-related activation in healthy volunteers.

**Results:**

Under-activations and over-activations were reported in a wide diversity of brain regions. Both under- and over-activations were significantly more likely to be located in hub nodes that constitute the “rich club” or core of the normative connectome. In a subset of 121 studies that reported both under- and over-activations in the same patients, we found that, in network terms, these abnormalities were located in close topological proximity to each other. Under-activation in a peripheral node was more frequently associated specifically with over-activation of core nodes than with over-activation of another peripheral node.

**Conclusions:**

Although schizophrenia is associated with altered brain functional activation in a wide variety of regions, abnormal responses are concentrated in hubs of the normative connectome. Task-specific under-activation in schizophrenia is accompanied by over-activation of topologically central, less functionally specialized network nodes, which may represent a compensatory response.

## Introduction

Functional neuroimaging studies in schizophrenia have identified alterations in a wide variety of brain regions and across cognitive domains. Frequently used tasks have explored executive functions,^1^ memory^2^ or emotional responses in patients,^3^ but also theory of mind,^4^ salience processing^5^ or time perception,^6^ among others. Furthermore, compared to controls, patients can exhibit either reduced activation, greater activation,^7,8^ or a combination of both,^9^ depending on the task and the regions concerned. Finding a common mechanistic explanation for this diversity of functional abnormalities is therefore challenging.^10^ Assessing the findings in the context of normal brain functional networks provides a potentially useful way of addressing this issue.

Historically, altered activation has been interpreted in the context of maps of neuroanatomical regions.^11^ This approach has contributed to “regional” interpretations of altered activation in schizophrenia,^12^ such as the notion of “hypofrontality”.^13,14^ An alternative approach involves interpreting abnormal activation in terms of disrupted interactions between regions, as in the “dysconnectivity” hypothesis of schizophrenia.^15–17^ This perspective highlights the extent to which brain regions are interconnected, forming a complex network.^18,19^ Arguably, these 2 approaches could inform each other. For example, we recently showed that in several brain disorders, including schizophrenia, the probability of finding a structural abnormality in a region depends on its degree of connectivity,^20^ with high-degree hub nodes having significantly increased probability of gray matter abnormality.

In the present study we used a similar network approach, but rather than focusing on structural abnormalities, we related abnormal functional activations in schizophrenia with the normal functional network configuration of the brain. Our purpose was 2-fold: to examine where abnormalities occur in schizophrenia in terms of their network position, and to explore the network relationship between under- and over-activations. We extracted the stereotactic coordinates of task-related under-activations and over-activations in patients compared to controls from 314 functional neuroimaging studies. The coordinates were then mapped onto a normative functional network, built from a separate meta-analysis of 1641 functional neuroimaging studies in healthy subjects.^21^ We thus examined the regions that were either under- or over-activated, as well as their relationship, in terms of their topological (network) characteristics. Our first hypothesis was that under-activation in schizophrenia would be widely distributed across the brain, but particularly concentrated in regions that are highly connected. Our second prediction was that in network terms, over-activations would be topologically close to under-activations (ie, separated by a short network path), supporting the view that the former may represent a compensatory response to reduced activation of the latter.^1^

## Methods

### Meta-Data

Our aim was to include all published task-based functional neuroimaging studies reporting coordinates of significant activation differences in patients with schizophrenia compared to healthy controls in Pubmed and BrainMap database.^22–24^ Details of the search and exclusion criteria can be found in the supplementary information.

A study typically included several “contrasts” between different task conditions. For example, in an *N*-back working memory paradigm, the contrasts might include 2-back vs 0-back, or 2-back vs 1-back. Furthermore, between-group contrasts might examine whether patients activated less than controls (“under-activations”) or more than controls (“over-activations”) during these tasks. We extracted the coordinates of significant between-group differences for all the contrasts reported in the included studies (whole-brain analyses at *P* < .001 uncorrected or less).

We grouped studies into 21 different paradigm classes according to the tasks used, based on the Brainmap taxonomy^22,23^ (supplementary table S1).

### Brain Parcellation

We divided the brain into 638 regions of interest (ROIs; described in ref.^21^). Regions were created from subdivisions of the Automated Anatomical Labeling Atlas^25^ following established methods.^26^ The resulting ROIs respected anatomical boundaries (ie, would not span between hemispheres) and were similarly sized in order to avoid bigger ROIs accumulating more differential activations. The size of the ROIs was tuned to correspond to the way activations were modeled in previous studies.^27,28^ Coordinates of regions with differential activation between groups were mapped onto this parcellation.

### Analyses in Anatomical Space

We first calculated the probability that a contrast in a study would report a differential activation (under and over-activations separately) for each ROI:

$$p(r)=\frac{n}{N}$$ (1)

where *n* is the number of contrasts reporting a differential activation in region *r* across *N* contrasts.

To account for the over-representation of certain tasks in the literature, we calculated these probabilities separately for studies grouped within the same paradigm class, and then averaged these group probabilities across classes. A few studies included tasks seldom used in the literature, which could lead to a noisy estimate. We therefore included in a balanced and domain-general analysis all tasks used in more than 5% of all studies. We also present domain-specific results, including the pooled group of the remainder of studies that did not reach the 5% threshold.

### Network Template (“Normative Connectome”) and Network Analyses

**Fig. 1.**
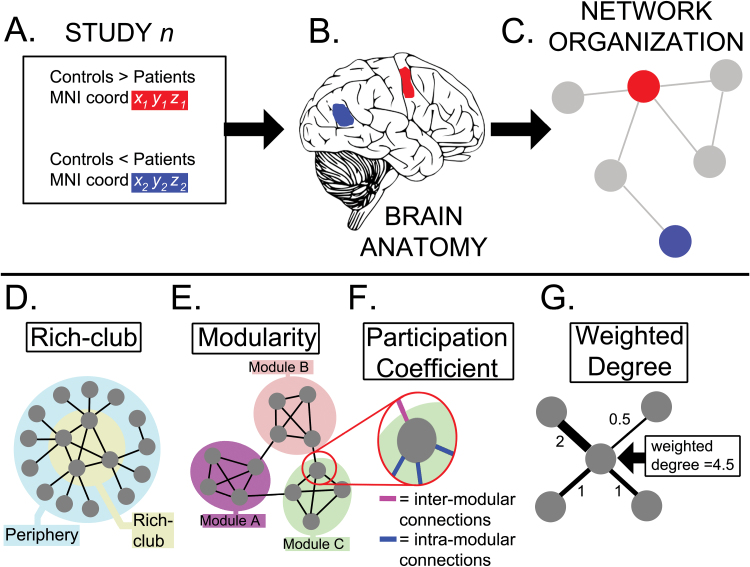
Overview of the methods and terminology. (**A**) The coordinates of under-activations (red) and over-activations (blue) during a task in schizophrenia were retrieved. (**B**) These were mapped onto regions of interest (ROIs) defined in standard neuroanatomical space. (**C**) The coordinates were also mapped onto a normative connectome (brain network) based on the same ROIs. This network “mapping” exercise provided information about the position in the network where each differential activation was located. We were particularly interested in the relationship between abnormal activations and specific network configurations such as the *rich club* (highly connected high degree nodes) (**D**) or network *modules* (groups of densely connected nodes that are sparsely connected to nodes in other modules) (**E**). We also examined their association with the *participation coefficient* (**F**), which refers to the relative proportions of intra-modular and inter-modular connections a node mediates, and the *weighted degree* (**G**), which is the sum of connections to a node.

We then related certain network characteristics of a region to functional abnormalities (figure 1). This approach is frequently used in social network analysis, where properties of the relationship ties between individuals (such as friendship) are related to variables describing the subjects (such as socioeconomic status).^29^

To inform the network characteristics of the regions included, we used a previously published functional brain network extracted from 1641 task-based neuroimaging studies of healthy subjects.^21^ This network was built by examining co-activation patterns across a wide range of tasks as a measure of functional connectivity,^27^ and used the same regional parcellation as described above. The resulting network was sparse, modular, with a rich-club organization, and a heavy-tailed degree distribution with hubs located in fronto-parietal regions; see ref.^21^ for details. We selected this network template because it was built from the same type of data as acquired in the studies involving patients with schizophrenia.

Regions of the parcellation where abnormal activations were located were labeled with several normative network properties. We explored whether the probability of an abnormal activation was related to the modular organization of the network,^30^ the existence of a rich-club,^31^ or the weighted degree^32^ and partition coefficient^33,34^ of a region. A description of these metrics is included in figure 1 and the supplementary methods.

Under- and over-activations can be observed during the same task. Furthermore, they might be related, as has been proposed in terms of the compensatory role of over-activations. To examine this possible link, we explored whether over-activated regions had a non-random topological relationship to under-activated regions. We therefore analyzed the subset of studies (*n* = 121) that reported under- and over-activations in the same sample and in response to the same task. The lower number of studies prevented us from doing a balanced analysis for different tasks, so we only present pooled results. For each of these studies, we assessed the following properties of all possible dyads (or pairs) of under- and over-activated locations:

- Physical distance: the Euclidean distance between centroids of the pair of regions.
- Topological distance: the shortest path existing between them in the network template.
- Similarity of their connectivity fingerprint^35^:

$$J(A,B)=\frac{A_{\text{target}}\cap B_{\text{target}}}{A_{\text{target}}\cup B_{\text{target}}}$$ (2)

where *A*_target_ and *B*_target_ refer to the regions connecting to regions *A* and *B* respectively (excluding connections between *A* and *B*). This is a metric measuring the similarity between the pattern of connections of 2 nodes.

- Proportion of intramodular dyads: both under and over-activations occuring in the same module.
- Proportion of within or between core/periphery dyads.

We then averaged the properties of the dyads within each study, and compared them to a null model.

### Alternative Normative Connectome

Hubs in the co-activation normative connectome are frequently co-active with other regions in many tasks, and are therefore the most frequently reported activations in the literature. Thus, a possible over-representation of abnormal activations in co-activation hubs could be due to a sampling bias. To exclude this possibility, we examined the relationship between centrality and functional abnormalities using an alternative connectome based on resting-state functional MRI from 27 healthy controls, described in a previous publication.^21^ Considering the possible bias of degree as a measure of “hubness” in resting-state functional networks, we used the participation coefficient.^36^

### Statistical Analyses

All analyses were statistically tested using permutation tests. For each comparison we built a null distribution where activations, their probability, or network labels were randomly permuted in the brain template (10 000 iterations). For example, the null model testing the relationship between probability of an under-activation in schizophrenia and the degree of a region was built by randomly permuting the degrees of regions and then calculating their correlation with under-activations. For the dyad analysis, only over-activations were randomly permuted among brain regions. This ensured that our null model would control for the degree of the under-activated brain region, which is particularly important if (as hypothesized) these were hubs. Two-tailed *P* values were inferred from comparing parameters in the data to the equivalent parameters in the null models, with *P* < .05 considered significant. Further details can be found in the supplementary methods.

## Results

### Task-Related Functional Studies of Schizophrenia

**Fig. 2.**
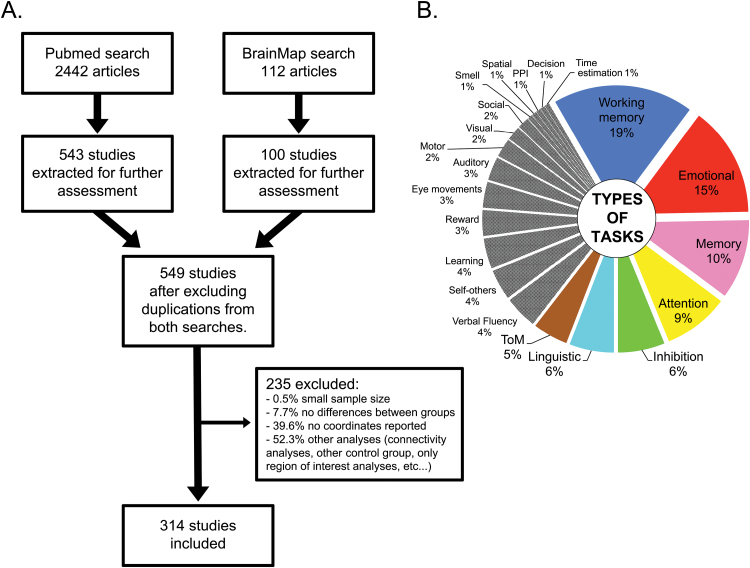
Literature search and studies included. (**A**) Flow-chart of literature search. (**B**) Characteristics of paradigms in included studies. In color, tasks that were present in more than 5% of publications. These were balanced in the domain-general analysis.

We included 314 task-based functional neuroimaging studies (figure 2A), comprising 723 different contrasts on 5291 patients with schizophrenia and 5651 controls. Detailed characteristics of the studies can be found in supplementary figure S1 and supplementary table S1.

The most frequent types of tasks used (more than 5% of the total; figure 2B) were working memory (19% of studies), emotion (15%), other forms of memory (10%), attention (9%), inhibition (6%), language (6%), and theory of mind (5%). Working memory tasks were over-represented in the schizophrenia literature compared to the non-schizophrenia literature, while linguistic studies were under-represented. A detailed comparison between tasks used in schizophrenia and healthy volunteers, and changes over time in the frequency with which different types of task have been the focus of imaging studies in schizophrenia, is included in the supplementary results and supplementary figure S2.

### Anatomical Analysis of Abnormal Activations

**Fig. 3.**
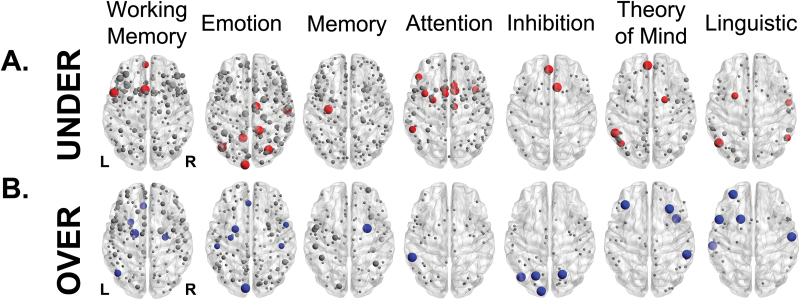
Anatomical location of under- and over-activations in schizophrenia elicited by different tasks. Regions sized according to the probability of finding an under-activation (**A**) or an over-activation (**B**) in the most frequently reported tasks. Nodes in red/blue represent locations where differential activations were statistically more frequent than the null model (*P* < .01, uncorrected), and grey nodes where they did not reach statistical significance. Across different tasks, there was no consistency in the anatomical distribution of differential activation.

We first examined whether under-activations (activations in patients < controls) tended to occur in certain neuroanatomical regions across different tasks, which would support the notion that the pathophysiology of schizophrenia involved localized dysfunction in particular regions, such as the dorsolateral prefrontal cortex.^12–14^ We found that across tasks, under-activations were widely distributed across the brain, with 82% of brain regions being the location of at least 1 under-activation. In line with previous reports^1–3^ and consistent with the notion that the topographical heterogeneity of differential activation is dependent on the type of task used, domain-specific meta-analyses provided evidence for the concentration of under-activations in specific regions (figure 3A, regions reported at *P* < .01 uncorrected for multiple comparisons, permutation tests. Detailed results for all tasks can be found in the supplementary results). Only 2 ROIs were sites of under-activations in more than 1 task: the right middle cingulum and the right thalamus. This limited overlap across tasks provided little support for the existence of a core localized brain abnormality in schizophrenia, because its extent was not statistically different from that observed after randomly permuting under-activations across regions in each task (*P* = .12, permutation test).

Similarly, over-activations in patients across tasks (activations in patients > controls) were also widely distributed, with 70% of brain regions being the location of at least 1 over-activation. Individual analyses of specific tasks showed a pattern of localized abnormalities (figure 3B and supplementary results). There was no anatomical overlap between regions across tasks that were the sites of task-specific over-activation.

### Network Analysis of Abnormal Activations

We then mapped under- and over-activations defined in the coordinates of standard anatomical space onto the normative functional neuroimaging co-activation graph.^21^

**Fig. 4.**
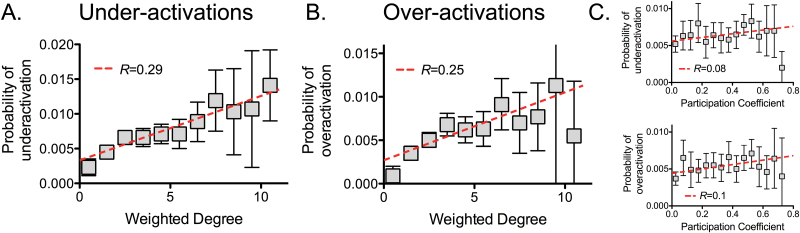
Centrality and probability of an abnormal activation. Probability of an (**A**) under- and (**B**) over-activation across tasks, and weighted degree in the functional co-activation connectome (with 95% confidence intervals and fitted regression line). A higher weighted degree indicates a more connected node. (**C**) Probability of an under- and over-activation across tasks and centrality defined by the participation coefficient of a normative connectome based on resting-state functional magnetic resonance imaging (fMRI) in healthy controls.

The probability of regional under-activation, in a domain-general analysis balanced for type of task, was significantly correlated with the weighted degree of the anatomically corresponding node in the normative connectome (figure 4A; *R* = 0.29, *P* < 10^−5^ permutation test). The relationship was also present when considering participation coefficient^36^ as the measure of network centrality, albeit to a lesser degree (*R* = 0.11, *P* < .002). Similarly, under-activations were significantly more likely to be located in rich-club nodes than in peripheral nodes of the normative connectome (1.83 times more likely, *P* < .003, permutation test; supplementary figure S3A). The relationship between normative weighted degree and probability of under-activation was not driven by one type of task, but was evident across different tasks, as shown by domain-specific analyses focused on working memory, emotional, attention and linguistic tasks (*P* < .05, permutation test; supplementary figure S4A).

Likewise, the probability of over-activation, in a balanced domain-general analysis, was positively correlated with normative weighted nodal degree (figure 4B; *R* = 0.25, *P* < 10^−5^, permutation test); again, this relationship was recapitulated in domain-specific analyses for working memory, attention, inhibition, memory, and language tasks (supplementary figure S4B). Over-activations were also concentrated in rich-club nodes in a domain-general analysis (1.58 times more likely, *P* < .04, permutation test; supplementary figure S3A). However, the correlation between probability of over-activation and the participation coefficient was not significant (*R* = 0.05, *P* = .22), suggesting that the relationship with weighted degree was driven by intramodular hubs.

The community structure of the normative connectome was decomposed into 4 modules, which were activated in response to specific tasks (supplementary figure S3B).^21^ In a balanced domain-general analysis, no single module was associated with a higher concentration of under-activations. In contrast, over-activations were not uniformly distributed across modules in the domain-general meta-analysis (*P* < .03, permutation test), with an over-representation of over-activations in the fronto-parietal module (supplementary figure S3B). The fronto-parietal module was previously shown to contain most of the rich-club nodes of the normative connectome.^21^

### Network Relationship Between an Over-Active and an Under-Active Region

We then analyzed the subgroup of 121 primary studies that reported under- and over-activations in the same sample performing the same task. We identified dyads composed of a region presenting an under-activation coupled with another region presenting an over-activation, and explored their network relationships.

**Fig. 5.**
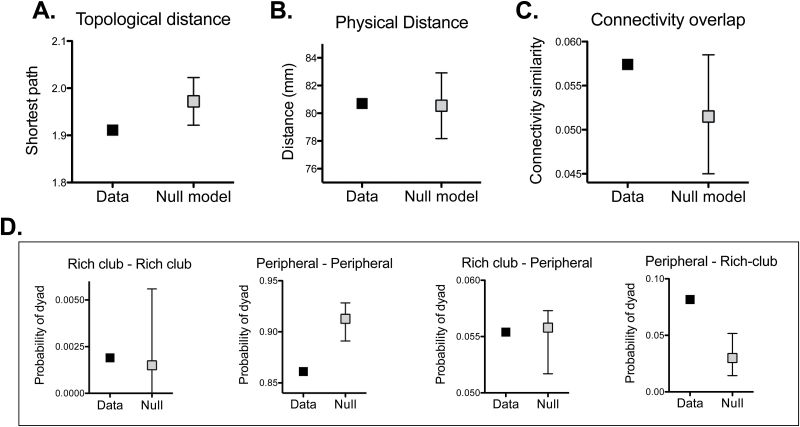
Characteristics of dyads of under- and over-activated regions in response to the same task. Topological distance (**A**), physical distance (**B**), and similarity between connectivity profiles (**C**) between pairs of under- and over-activated regions. 95% confidence intervals shown. (**D**) Post hoc analyses exploring frequencies of the observed dyads and the rich-club/periphery configuration. Graphs are named according to type of region under-activated/ over-activated.

The topological distance (shortest path) between under- and over-activated regions was shorter than expected by chance (*P* < .007, permutation test; figure 5A). In contrast, the physical (Euclidean) distance between the 2 regions was not (*P* = .55, permutation test; figure 5B). The 2 nodes within a dyad of over- and under-activations had a similar connectivity profile (ie, both nodes connected to a similar group of regions of the brain). However, this was not significantly different from the similarity observed between connectivity profiles in randomly drawn pair of nodes (*P* = .08, permutation test; figure 5C). In relationship to the community structure of the connectome, dyadic pairs were not significantly more likely to be located in the same module (*P* = .23, permutation test). However, dyads were not randomly distributed with respect to the core-periphery organization of the brain network (*P* < 10^−4^, permutation test). There was significant over-representation of dyads comprising an under-activated peripheral node and an over-activated rich-club node, and a significant under-representation of dyads comprising an under-activated peripheral node coupled to another over-activated peripheral node (figure 5D).

### Analyses of Centrality Defined Using an Alternative Connectome

We finally looked at the relationship between centrality and abnormal activations using an alternative normative connectome built from resting-state functional magnetic resonance imaging (fMRI) in healthy controls as a way to exclude a sampling bias. A significant (albeit weaker) correlation between the balanced domain-general probability of an under-activation and centrality was also present (*R* = 0.08, *P* < .045; figure 4C). Likewise, the relationship was present for over-activations (*R* = 0.10, *P* < .022; figure 4C).

## Discussion

The functional neuroimaging literature in schizophrenia comprises a potentially confusing array of findings that include most regions of the brain. We employed a novel approach to interpreting these findings that involved a meta-analysis of 314 neuroimaging studies of patients with schizophrenia combined with a normative functional connectome. In this way, we sought to leverage the power of the aggregated functional neuroimaging literature, and use information on normal brain network topology to understand the results.

In line with our first hypothesis, both under and over-activations in schizophrenia were widely distributed across the brain. This is consistent with the global nature of brain dysfunction in schizophrenia, and has been discussed in both the neuroimaging^37^ and neuropsychological literature.^38^ Our results add further weight to the argument that it is unlikely that schizophrenia can be fully understood in terms of an anatomically localized abnormality of brain function such as reduced prefrontal activation (hypofrontality), or abnormal co-activation of fronto-temporal or fronto-striatal systems.

Topological analysis of differential activation in schizophrenia showed that under-activations were concentrated in hubs, and the rich clubs that these hubs form. This echoes previous studies that have implicated hubs in the pathophysiology of schizophrenia^39,40^ and other brain disorders,^41^ and with evidence that structural abnormalities in brain disorders, including schizophrenia, are preferentially located in hubs.^20^ Moreover, network analyses of resting-state fMRI have shown a reduced probability of identifying high-degree hubs in patients with schizophrenia than in controls.^42,43^ As such, our data and others support the notion that normal connectome topology is an important constraint on the distribution of regional abnormalities in schizophrenia and other brain disorders.^44^

A novel insight stemming from our current analysis of functional studies, which was not evident in our previous structural analysis, is that the set of abnormally activated hubs varied according to the task performed. This does not support a localizationist perspective on the pathology of schizophrenia, or an absolute dysfunction of hubs. Instead, it suggests that schizophrenia could be related to a generalized decrease in hub capacity. From a molecular perspective, this is consistent with data from genetic,^45,46^ psychopharmacological,^47^ and neurochemical imaging studies^48^ implicating glutamatergic synaptic abnormalities in schizophrenia. Although glutamatergic synapses are present throughout the brain, particularly in long inter-regional projections, a dysfunction in glutamatergic transmission would be likely to have most impact in regions with the highest concentration of such connections (ie, hubs).

Our analysis of under- and over-activations during the same task revealed a higher frequency of over-activation in high-degree regions, particularly when this was accompanied by an under-activation in a peripheral node. Topologically central regions are, by definition, closer to all other brain regions, and their connection profile might overlap with many peripheral nodes due to their high number of connections. Studying over-activations alongside under-activations showed that the pairs were indeed closer in network terms, with a trend-level similarity of their respective connection profiles. In line with previous suggestions,^1,49^ one interpretation of these results would be that recruiting topologically central nodes is an appropriate response of a system that is trying to compensate for a failing, peripheral region. In other words, because hubs are in a central position in the network where they are close to all regions, and have a similar connection profile to many other nodes, they are ideally placed to “step in” and support a failing region if needed. This is consistent with the notion that hubs are less cognitively specialized than peripheral nodes,^21^ with the capacity to mediate several different tasks if required.

The distribution of cognitive tasks in the neuroimaging literature in schizophrenia is heterogeneous. Although we tried to limit its impact by pooling sub-groups of similar tasks, it was not possible to include data relating to cognitive processes that have not been examined in previous studies. Most studies involved chronically ill and medicated patients, so we cannot exclude the effects of illness duration and treatment on our results. As in other brain imaging meta-analytic approaches,^50^ we did not include studies that did not find significant between-group differences. The way functional abnormalities are modeled in our network approach may have influenced the findings. For example, we did not model the effect of sample size on the precision of the coordinates, and activations from cognitive contrasts reported from a single study were treated similarly to contrasts from different studies, which does not take into account the lower variance in data from within a single study. While we used another network template based in resting-state fMRI to corroborate some of our findings, we did not explore the effect of using other parcellation schemes with larger or smaller ROIs.^26^ Finally, task performance data was not always available, and its inclusion would have helped characterize abnormal activations.^7^

In conclusion, although schizophrenia is associated with altered neural responses in a wide variety of brain regions, abnormal activation appears to be concentrated in task-specific hubs. Our network analysis of over-activations was compatible with the notion that regional over-activation may represent a compensatory response to under-activation elsewhere.

## Supplementary Material

Supplementary material is available at http://schizophreniabulletin.oxfordjournals.org

## Funding

Research Training Fellowship from the Wellcome Trust (WT093907AIA to N.A.C.); Medical Research Council (United Kingdom) and the Wellcome Trust (to the Behavioural and Clinical Neuroscience Institute); National Institutes of Health/ National Institute of Mental Health (R01 MH074457 to BrainMap).

## Supplementary Material

Supplementary Data
