# Supplementary material for: Altered Hub Functioning and Compensatory Activations in the Connectome: A Meta-Analysis of Functional Neuroimaging Studies in Schizophrenia
Source: Schizophr Bull. 2015 Oct 15;42(2):434–42. doi: 10.1093/schbul/sbv146 (PMC4753609; doi:10.1093/schbul/sbv146)
Supplement: Supplementary Data [file supp_42_2_434__index.html]

Altered Hub Functioning and Compensatory Activations in the Connectome: A Meta-Analysis of Functional Neuroimaging Studies in Schizophrenia — Altered Hub Functioning and Compensatory Activations in the Connectome: A Meta-Analysis of Functional Neuroimaging Studies in Schizophrenia — Supplementary Data 

# Altered Hub Functioning and Compensatory Activations in the Connectome: A Meta-Analysis of Functional Neuroimaging Studies in Schizophrenia

## Supplementary Data

Data files

- Supplementary Data - Supplementary Data
- Supplementary Data - Supplementary Data
- Supplementary Data - Supplementary Data
