# Supplementary material for: Altered Hub Functioning and Compensatory Activations in the Connectome: A Meta-Analysis of Functional Neuroimaging Studies in Schizophrenia
Source: Schizophr Bull. 2015 Oct 15;42(2):434–42. doi: 10.1093/schbul/sbv146 (PMC4753609; doi:10.1093/schbul/sbv146)
Supplement: Supplementary Data [file supp_sbv146_Supplementary_information_1.doc]

***Supplementary information***

**Altered hub functioning and compensatory activations in the connectome: a meta-analysis of functional neuroimaging studies in schizophrenia**

N.A. Crossley, A. Mechelli, C. Ginestet, M. Rubinov, E.T. Bullmore and P. McGuire.

*1. Supplementary Methods*

*A. Literature search strategy and exclusion criteria*

*B. Graph analyses metrics*

*C. Statistical inferences from permutation tests*

*2. Supplementary Results*

*A. Comparison between schizophrenia and non-schizophrenia tasks used in the literature.*

*B. Anatomical location of under- and overactivations according to tasks.*

*3. Supplementary Figures*

*A. Figure S1: Characteristics of studies, contrasts, and subjects included.*

*B. Figure S2: Characteristics of the most commonly used tasks in the schizophrenia literature.*

*C. Figure S3: Under and over-activations in schizophrenia and the community structure of the coactivation network.*

*D. Figure S4: Relationship between the degree of a region and the probability of abnormal activation in specific tasks.*

*4. Supplementary Table*

*A. Table S1: Studies included.*

*5. References*

***1. Supplementary Methods***

***A. Literature search strategy and exclusion criteria***

We aimed to include all relevant studies published in English language in Pubmed and BrainMap databases. The Pubmed search (7^th^ June'13) included the terms: 'functional MRI', 'fMRI', 'functional magnetic', 'PET', 'Positron emission', 'SPECT' and 'schizophreni*'. For the BrainMap database, all functional studies including patients with schizophrenia were retrieved (7^th^ June'13).

We excluded studies that examined genetic interactions, did not compare a task to a baseline, included less than 5 subjects in a group, included non-healthy subjects as the sole control group, or reported connectivity or non-voxel level analyses.

***B. Graph analyses metrics***

- *Centrality:* a concept describing the position of a node in a network, whether it is 'central' or 'peripheral'. There are several parameters measuring it, including degree, weighted degree, betweenness centrality, and participation coefficient.

- *Degree*: number of significant connections that a node has. This is a simple measure of how central a node is in a network.

- *Weighted degree* (strength): nodal characteristic that describes the sum of all the weights of its connections. Another measure of centrality in a network, highlighting the strength of a connection alongside the number of connections. This measure was used in the normative connectome, it is easy to interpret, and therefore we chose it as the main parameter to explore centrality in our study.

- *Density of a graph*: Percentage of existing edges in the network compared to all possible edges.

- *Shortest path*: the shortest topological distance (geodesic) between two nodes. The shortest path of a network is the average of all the shortest paths between all pairs of nodes. λ is the ratio between the shortest path of the network and comparable random networks (same number of nodes and edges, same degree and weight distribution) [^1^](#_ENREF_1).

*- Modularity*: the best partition of the network into subgroups which are highly connected between themselves. This is obtained by maximising a parameter *Q* [^2^](#_ENREF_2):

|  | 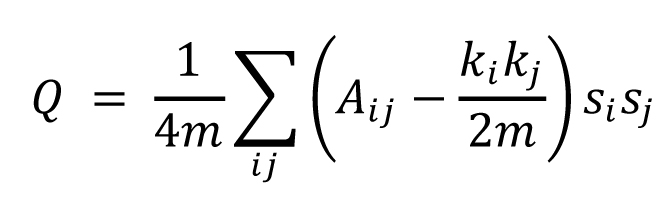 | [ S1 ] |
| --- | --- | --- |

where *A_ij_* is the edge between nodes *i* and *j*, with degrees denoted *k_i_* and *k_j_*, *m* denotes the total number of edges of the network, and *s_i_s_j_* is 1 if the nodes belong to the same group or -1 if not.

- *Participation coefficient*: another measure of centrality, which takes into account the proportion of connections that a node has with nodes from other modules [^3^](#_ENREF_3). Defined as:

|  | 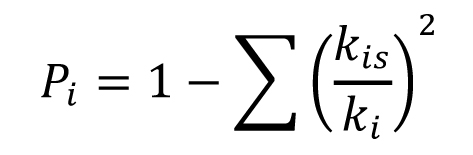 | [ S2 ] |
| --- | --- | --- |

where *k_is_* are the sum of the connections from node *i* to module *s*, and *k_i_* its degree. Thus, it measures how important a node is for inter-modular communication.

*- Rich-club coefficient*: describes whether high degree nodes in a network tend to be highly connected between themselves. In other words, after accounting for the larger number of connections of hubs, we measured whether they were still more likely to connect with other hubs. We here used its formulation for weighted networks, which is the proportion of the strongest edges of the network that connect high degree nodes. Formally, this is defined for a cut-off richness factor *r* as [^4^](#_ENREF_4):

|  | 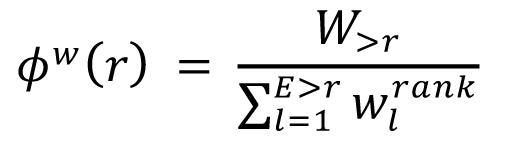 | [ S3 ] |
| --- | --- | --- |

where *W_>r_* is the sum of the weights of the connections between nodes with a richness factor higher than *r*, *E>r* the number of these edges, and the denominator describes the sum of the top *E>r* strongest edges of the network. We here used the weighted degree as the richness factor to rank the nodes.

***C. Statistical inferences from permutation tests***

As described in the main text, we performed permutation analysis to provide a statistical inference to every test reported here. The general approach was to build a null model from 10,000 permutations of the original data, and then compare a parameter in the observed data to this distribution, building a *P* value (two-tailed reported). We here provide more detail for each analysis:

- *Anatomical distribution of abnormal activations*: for each study included, we randomly permuted the reported activations in the brain template. For each region we then calculated its probability of abnormal activation.

- *Network properties of the reported abnormalities*: to provide statistical inference to network properties, we randomly permuted the network labels assigned to each region (e.g., degree, module assignment or rich-club/periphery label). In the case of degree, we then compared the correlation between degree and probability of the observed and null model. For the rich-club organisation, we measured the difference of the average probability of an abnormal activation in a rich-club node and a peripheral node in the observed and null model. For the modular organisation (4 modules), we measured the deviation of the modules' probabilites to the expected whole-brain probability using the following parameter *D*:

|  | 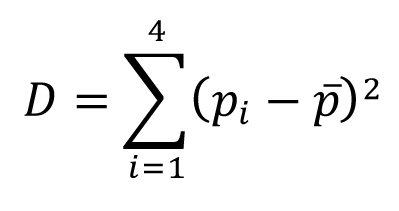 | [ S4 ] |
| --- | --- | --- |

where *p_i_* is the probability of abnormal activation of module *i*, and
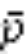
 the whole-brain probability. The observed *D* was compared to the null model *D*.

- *Network analysis of dyads*: we here only randomly permuted the overactivations of the dyads, excluding the case where the random permutation resulted in the same overactivations as the observed ones. We then compared the specific graph analytic metrics (shortest path, Euclidean distance, connectivity profile), the probability of an intramodular dyad (as opposed to an intermodular), and the probability of dyads being distributed non-trivially according to the network's rich-club/peripheral organization. For the latter, we used a similar metric to the modularity *D* parameter described above, comparing the probabilities of dyads entirely within rich-club nodes, peripheral nodes, or in between them.

***2. Supplementary Results***

***A. Comparison between schizophrenia and non-schizophrenia tasks used in the*** ***literature.***

We also looked at the differences between the types of tasks used in functional neuroimaging studies in schizophrenia and those used in healthy controls. This comparison provides hints about the predominating hypotheses about schizophrenia in the research community. On the other hand, the normative connectome used in this approach was built from the functional neuroimaging literature in healthy controls. We previously showed that the main properties of the normative connectome were unchanged when controlling for the over-representation of certain tasks in the literature [^5^](#_ENREF_5). However, the heterogeneity of studies could still confound some of the findings in our current analyses.

The seven most frequently used tasks in schizophrenia accounted for 71% of all the included studies. This was a significantly higher proportion than the one found in the overall BrainMap database of 58%, which could not be accounted by random error (*P*<10^-5^, as determined from random sampling). Within this group of over-represented studies in schizophrenia, there were also some differences. **Fig. S2A** provides detailed information about each specific task in schizophrenia, and its frequency in the non-schizophrenia literature. The frequency in schizophrenia of several tasks in this selected group diverted from random sampling. This was particularly noticeable in an over-representation of working memory tasks (2.4 times more frequently than expected), task which has been historically at the center of our understanding of schizophrenia [^6^](#_ENREF_6). More surprisingly was the lower frequency of linguistic tasks in schizophrenia studies (2.6 times less than expected), perhaps a cognitive domain where there is still no dominant task that could be used in a widespread manner in pathological conditions. Interestingly, both working memory and linguistic tasks did not show a great temporal fluctuation across years (**Fig. S2B**).

Overall these results suggest that tasks used in schizophrenia are not a representative sample of the cognitive studies in the whole literature. It is therefore unlikely that our results are driven by a shared bias in the normative connectome and schizophrenia studies caused by a similar imbalance in study type.

***B. Anatomical location of under- and overactivations according to tasks.***

Underactivations in working memory tasks were most frequent in prefrontal regions; in emotion tasks, they were concentrated in thalamic and occipito-temporal regions; in episodic memory tasks they were concentrated in left hippocampus; in attention tasks, anterior and middle cingulate cortex and basal ganglia; in inhibition tasks, anterior and middle cingulate cortex; in linguistic tasks (mostly semantic reading), lateral temporal regions and left putamen; and in theory of mind tasks, medial prefrontal, lateral temporal cortical and right pallidum (**Fig. 3A**).

In working memory tasks, overactivations were concentrated in medial temporal and anterior cingulate cortex; in emotional tasks, left amygdala and hippocampus, left medial frontal region, left cuneus and bilateral parietal; in episodic memory tasks, right medial temporal; in attention, left supramarginal gyrus; in inhibition, parietal and occipital cortex; in theory of mind tasks, frontal regions and right supramarginal cortex; and in language tasks, bilateral frontal cortex and left putamen (**Fig. 3B**).
