# Supplementary material for: Altered Hub Functioning and Compensatory Activations in the Connectome: A Meta-Analysis of Functional Neuroimaging Studies in Schizophrenia
Source: Schizophr Bull. 2015 Oct 15;42(2):434–42. doi: 10.1093/schbul/sbv146 (PMC4753609; doi:10.1093/schbul/sbv146)
Supplement: Supplementary Data [file supp_sbv146_Supplementary_information_2.doc]

***3. Supplementary Figures***

**Figure**

**Figure S1**

**
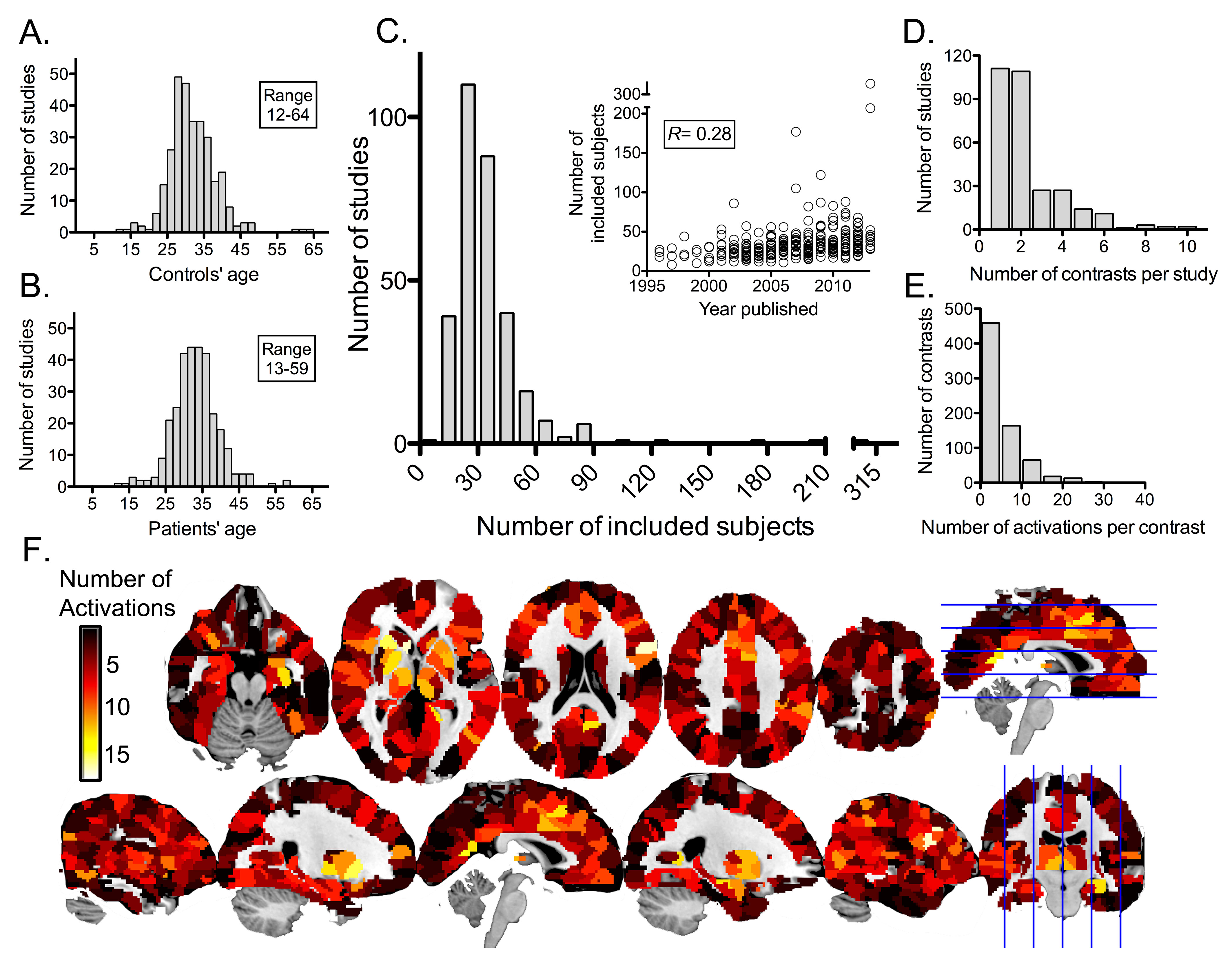
**

**Fig. S1. Characteristics of studies, contrasts, and subjects included.** Most of the studies (97.8%) included groups of subjects with a mean/median age between 18 and 60 years old (**A** and **B**). The majority of studies had a sample size below 50 subjects (**C**), which has been increasing throughout the years (**C inset**). Most studies included a few comparisons or contrasts (**D**), with each contrast reporting only a few regions of differential activation between groups (**E**). (**F**) Number of total differential activations reported in each region of interest, in all included contrasts from all studies.

**Figure S2**

**
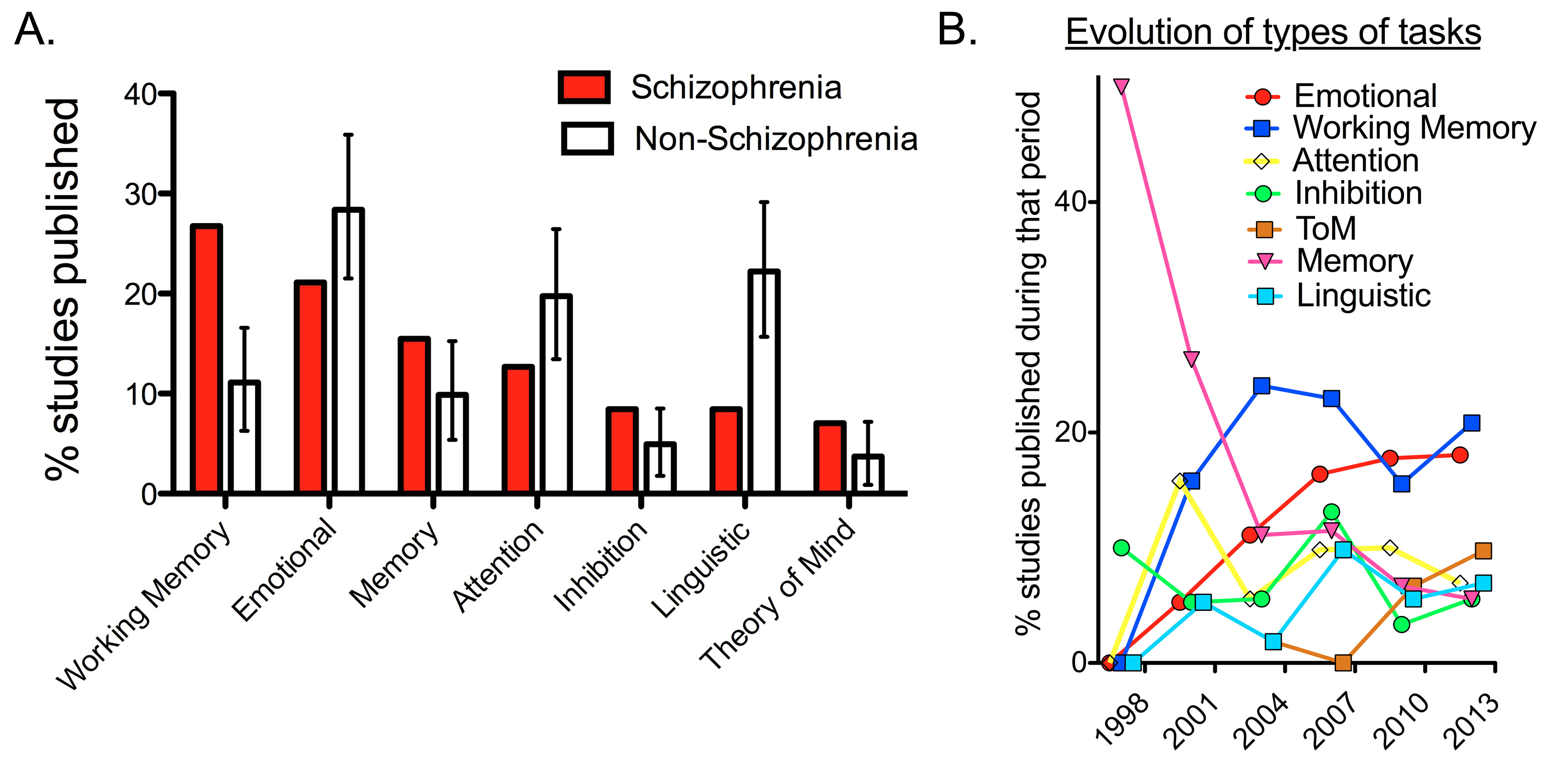
**

**Fig. S2. Characteristics of the most commonly used tasks in the schizophrenia literature. A)** Comparison of the frequency of the most common tasks used in the schizophrenia literature and the non-schizophrenia literature. Confidence interval shown corresponds to *P*<0.05 Bonferroni corrected, built from random sampling of the overall studies (10,000 iterations). Note that working memory tasks are over-represented in schizophrenia studies, while linguistic studies, and to a lesser extent emotion and attention tasks, are not. **B)** Changes over time of the relative frequency of different task domains used in schizophrenia, reflecting historical variations in the interests of the research community.

**Figure S3**

**
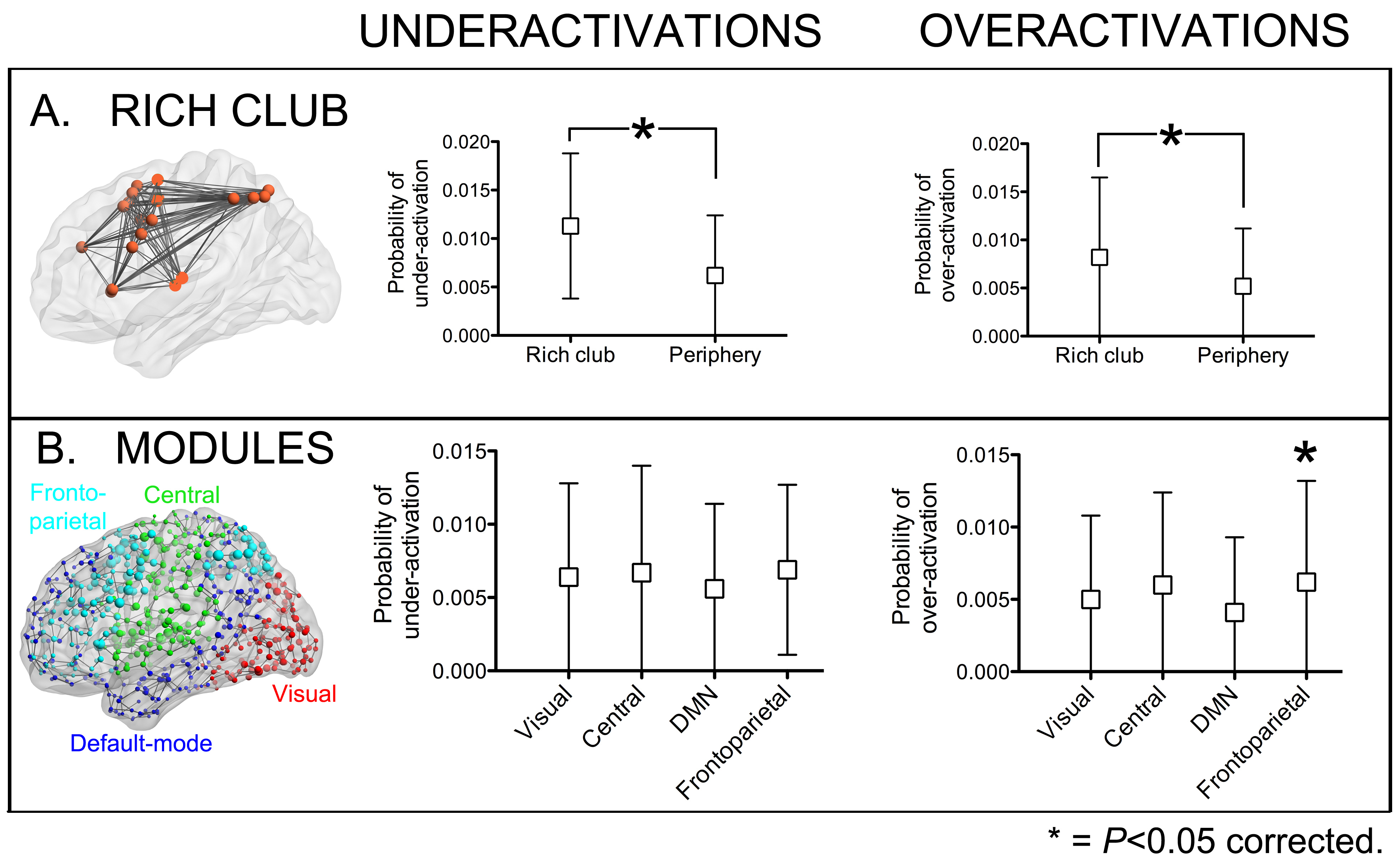
**

**Fig. S3**. **Under and over-activations in schizophrenia and the community structure of the coactivation network**. **A**) The brain coactivation network includes a highly connected group of hubs, or rich club. Both under- and over-activations are over-represented in the group of highly-connected regions. **B**) The brain coactivation network is also composed of highly connected regions forming modules, which are shown in neuroanatomical space. There was a significant excess of over-activations in the frontoparietal module.

**Figure S4**

**
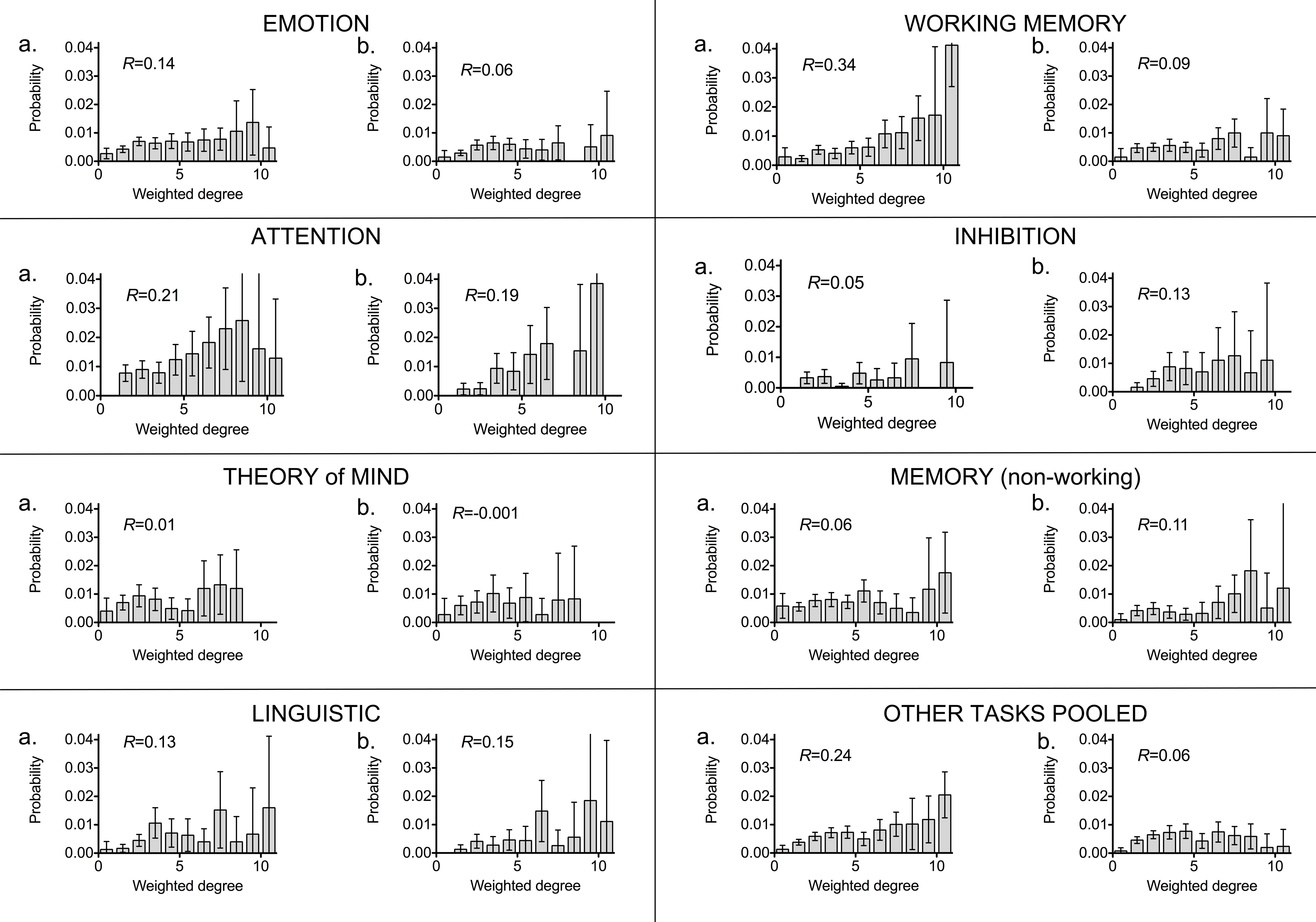
Fig. S4. Relationship between the degree of a region and the probability of abnormal activation in specific tasks.** Relationship between the weighted degree of a region and the probability of under-activation (described with an *a*) or over-activation (described with a *b*) in specific tasks, as well as in the pooled heterogeneous group of less frequently reported studies. (WM= working memory)
