# Supplementary material for: Altered Hub Functioning and Compensatory Activations in the Connectome: A Meta-Analysis of Functional Neuroimaging Studies in Schizophrenia
Source: Schizophr Bull. 2015 Oct 15;42(2):434–42. doi: 10.1093/schbul/sbv146 (PMC4753609; doi:10.1093/schbul/sbv146)
Supplement: Supplementary Data [file supp_sbv146_Supplementary_information_3.doc]

**Table S1 - Functional neuroimaging studies included: full list of references and their characterization**

|  |  |  |  |  |  |  |  |  |  |  |  |  |  |  |  |  |  |  |  |  |  |  |  | **Not from Brainmap taxonomy** | | |
| --- | --- | --- | --- | --- | --- | --- | --- | --- | --- | --- | --- | --- | --- | --- | --- | --- | --- | --- | --- | --- | --- | --- | --- | --- | --- | --- |
| **Year** | **First Author** | **Journal** | **Nr of patients / controls** | **Age of patients / controls ^** | **PMID** | Memory - working | Memory (non working) | Language - speech execution | Language | Attention | NoGo / Inhibition * | Wisconsin card sorting test | Audition | Vision | Olfaction | Space | Time | Motor execution | Saccades ** | Emotion | Theory of Mind *** | Self-reflection | Social | Reward | Pre-pulse inhibition | Decision under uncertainty |
| 2013 | Yoon | Biological Psychiatry | 18 / 19 | 33 / 29 | 23290498 | **1** |  |  |  |  |  |  |  |  |  |  |  |  |  |  |  |  |  |  |  |  |
| 2012 | Kyriakopoulos | J Am Acad Child Adolesc Psychiatry | 25 / 20 | 16 / 16 | 22917204 | **1** |  |  |  |  |  |  |  |  |  |  |  |  |  |  |  |  |  |  |  |  |
| 2012 | Sugranyes | Schizophrenia Research | 22 / 19 | 17 / 17 | 22475381 | **1** |  |  |  |  |  |  |  |  |  |  |  |  |  |  |  |  |  |  |  |  |
| 2012 | Dreher | Biological Psychiatry | 17 / 31 | 31 / 28 | 22341369 | **1** |  |  |  |  |  |  |  |  |  |  |  |  |  |  |  |  |  |  |  |  |
| 2011 | Thormodsen | Psychiatry Research: Neuroimaging | 15 / 15 | 16 / 16 | 22079661 | **1** |  |  |  |  |  |  |  |  |  |  |  |  |  |  |  |  |  |  |  |  |
| 2013 | Anticevic | Schizophrenia Bulletin | 28 / 24 | 36 / 37 | 21914644 | **1** |  |  |  |  |  |  |  |  |  |  |  |  |  |  |  |  |  |  |  |  |
| 2012 | Ragland | Neuroimage | 20 / 19 | 27 / 28 | 21907293 | **1** |  |  |  |  |  |  |  |  |  |  |  |  |  |  |  |  |  |  |  |  |
| 2011 | Foucher | Psychiatry Research: Neuroimaging | 17 / 17 | 30 / 30 | 21868203 | **1** |  |  |  |  |  |  |  |  |  |  |  |  |  |  |  |  |  |  |  |  |
| 2011 | Avsar | Psychiatry Research: Neuroimaging | 10 / 8 | 31 / 35 | 21782395 | **1** |  |  |  |  |  |  |  |  |  |  |  |  |  |  |  |  |  |  |  |  |
| 2011 | Ortiz-Gil | British Journal of Psychiatry | 49 / 39 | 41 / 40 | 21727234 | **1** |  |  |  |  |  |  |  |  |  |  |  |  |  |  |  |  |  |  |  |  |
| 2011 | Wolf | Neuropsychobiology | 8 / 8 | 27 / 28 | 21701227 | **1** |  |  |  |  |  |  |  |  |  |  |  |  |  |  |  |  |  |  |  |  |
| 2011 | Diaz | Journal of Psychiatric Research | 11 / 17 | 33 / 24 | 21411108 | **1** |  |  |  |  |  |  |  |  |  |  |  |  |  | **1** |  |  |  |  |  |  |
| 2011 | White | Schizophrenia Research | 22 / 24 | 15 / 15 | 21211946 | **1** |  |  |  |  |  |  |  |  |  |  |  |  |  |  |  |  |  |  |  |  |
| 2011 | Bor | Schizophrenia Research | 22 / 15 | 28 / 30 | 21067898 | **1** |  |  |  |  |  |  |  |  |  |  |  |  |  |  |  |  |  |  |  |  |
| 2011 | Krawitz | Neuroimage | 14 / 12 | 41 / 41 | 20851194 | **1** |  |  |  |  |  |  |  |  |  |  |  |  |  |  |  |  |  |  |  |  |
| 2010 | Kim | PLoS One | 12 / 13 | 40 / 40 | 20725639 | **1** |  |  |  |  |  |  |  |  |  |  |  |  |  |  |  |  |  |  |  |  |
| 2010 | Habel | The World Journal of Biological Psychiatry | 14 / 14 | 37 / 36 | 20642398 | **1** |  |  |  |  |  |  |  |  |  |  |  |  |  | **1** |  |  |  |  |  |  |
| 2010 | Schlagenhauf | Schizophrenia Research | 11 / 11 | 34 / 38 | 20189356 | **1** |  |  |  |  |  |  |  |  |  |  |  |  |  |  |  |  |  |  |  |  |
| 2010 | Luck | Hippocampus | 17 / 17 | 30 / 30 | 19693783 | **1** |  |  |  |  |  |  |  |  |  |  |  |  |  |  |  |  |  |  |  |  |
| 2009 | Henseler | European Journal of Neuroscience | 12 / 12 | 33 / 32 | 19686473 | **1** |  |  |  |  |  |  |  |  |  |  |  |  |  |  |  |  |  |  |  |  |
| 2009 | Royer | Psychiatry Research: Neuroimaging | 19 / 12 | 33 / 34 | 19643585 | **1** |  |  | **1** |  |  |  |  |  |  |  |  |  |  |  |  |  |  |  |  |  |
| 2009 | Hamilton | Human Brain Mapping | 20 / 38 | 32 / 33 | 19449330 | **1** |  |  |  |  |  |  |  |  |  |  |  |  |  |  |  |  |  |  |  |  |
| 2009 | Karch | Journal of Psychiatric Research | 61 / 61 | 38 / 39 | 19426993 | **1** |  |  |  |  |  |  |  |  |  |  |  |  |  |  |  |  |  |  |  |  |
| 2008 | Pae | International Journal of Neuroscience | 12 / 11 | 28 / 25 | 18788030 | **1** |  |  |  |  |  |  |  |  |  |  |  |  |  |  |  |  |  |  |  |  |
| 2008 | Pomarol-Clotet | Psychological Medicine | 32 / 32 | 42 / 41 | 18507885 | **1** |  |  |  |  |  |  |  |  |  |  |  |  |  |  |  |  |  |  |  |  |
| 2008 | Koch | Neuroscience | 41 / 41 | 30 / 29 | 18359576 | **1** |  |  |  |  |  |  |  |  |  |  |  |  |  |  |  |  |  |  |  |  |
| 2008 | Lee | PLoS One | 8 / 7 | 35 / 25 | 18335036 | **1** |  |  |  |  |  |  |  |  |  |  |  |  |  |  |  |  |  |  |  |  |
| 2007 | Haenschel | Archives of General Psychiatry | 17 / 17 | 18 / 18 | 17984392 | **1** |  |  |  |  |  |  |  |  |  |  |  |  |  |  |  |  |  |  |  |  |
| 2008 | Schlosser | Neuropsychologia | 41 / 41 | 30 / 29 | 17707869 | **1** |  |  |  |  |  |  |  |  |  |  |  |  |  |  |  |  |  |  |  |  |
| 2007 | Barch | American Journal of Psychiatry | 57 / 120 | 32 / 27 | 17606661 | **1** |  |  |  |  |  |  |  |  |  |  |  |  |  |  |  |  |  |  |  |  |
| 2008 | Scheuerecker | Journal of Psychiatric Research | 23 / 23 | 32 / 33 | 17559877 | **1** |  |  |  |  |  |  |  |  |  |  |  |  |  |  |  |  |  |  |  |  |
| 2007 | Schneider | Psychiatry Research | 13 / 26 | 33 / 33 | 17532193 | **1** |  |  |  |  |  |  |  |  |  |  |  |  |  |  |  |  |  |  |  |  |
| 2007 | Walter | Neuroimage | 15 / 17 | 33 / 31 | 17363277 | **1** |  |  |  |  |  |  |  |  |  |  |  |  |  |  |  |  |  |  |  |  |
| 2006 | Meisenzahl | European Archives of Psychiatry and Clinical Neuroscience | 12 / 12 | 34 / 34 | 17151834 | **1** |  |  |  |  |  |  |  |  |  |  |  |  |  |  |  |  |  |  |  |  |
| 2006 | Tan | American Journal of Psychiatry | 15 / 26 | 33 / 32 | 17074949 | **1** |  |  |  |  |  |  |  |  |  |  |  |  |  |  |  |  |  |  |  |  |
| 2007 | Schneider | Schizophrenia Research | 48 / 57 | 31 / 31 | 17010573 | **1** |  |  |  |  |  |  |  |  |  |  |  |  |  |  |  |  |  |  |  |  |
| 2006 | Kumari | Schizophrenia Research | 25 / 13 | 34 / 33 | 16616832 | **1** |  |  |  |  |  |  |  |  |  |  |  |  |  |  |  |  |  |  |  |  |
| 2006 | Johnson | Biological Psychiatry | 18 / 18 | 37 / 37 | 16503328 | **1** |  |  |  |  |  |  |  |  |  |  |  |  |  |  |  |  |  |  |  |  |
| 2006 | Camchong | Biological Psychiatry | 14 / 14 | 37 / 40 | 16458267 | **1** |  |  |  |  |  |  |  |  |  |  |  |  |  |  |  |  |  |  |  |  |
| 2005 | Tan | American Journal of Psychiatry | 11 / 11 | 25 / 26 | 16199831 | **1** |  |  |  |  |  |  |  |  |  |  |  |  |  |  |  |  |  |  |  |  |
| 2005 | Mendrek | Psychological Medicine | 12 / 12 | 29 / 28 | 15841676 | **1** |  |  |  |  |  |  |  |  |  |  |  |  |  |  |  |  |  |  |  |  |
| 2005 | Manoach | Schizophrenia Research | 16 / 12 | 42 / 35 | 16076549 | **1** |  |  |  |  |  |  |  |  |  |  |  |  |  |  |  |  |  |  |  |  |
| 2005 | Meyer-Lindenberg | Archives of General Psychiatry | 22 / 22 | 31 / 32 | 15809405 | **1** |  |  |  |  |  |  |  |  |  |  |  |  |  |  |  |  |  |  |  |  |
| 2005 | Yoo | International Journal of Neuroscience | 10 / 10 | 25 / 23 | 15804721 | **1** |  |  |  |  |  |  |  |  |  |  |  |  |  |  |  |  |  |  |  |  |
| 2005 | Thermenos | Schizophrenia Research | 14 / 22 | 38 / 38 | 15721998 | **1** |  |  |  |  |  |  |  |  |  |  |  |  |  |  |  |  |  |  |  |  |
| 2004 | Quintana | Psychiatry Research: Neuroimaging | 8 / 8 | 35 / 29 | 15598546 | **1** |  |  |  |  |  |  |  |  |  |  |  |  |  |  |  |  |  |  |  |  |
| 2004 | Mendrek | British Journal of Psychiatry | 8 / 8 | 30 / 28 | 15339824 | **1** |  |  |  |  |  |  |  |  |  |  |  |  |  |  |  |  |  |  |  |  |
| 2004 | Kindermann | Schizophrenia Research | 10 / 12 | 58 / 64 | 15099603 | **1** |  |  |  |  |  |  |  |  |  |  |  |  |  |  |  |  |  |  |  |  |
| 2004 | Jansma | Schizophrenia Research | 10 / 10 | 27 / 28 | 15099600 | **1** |  |  |  |  |  |  |  |  |  |  |  |  |  |  |  |  |  |  |  |  |
| 2004 | Jacobsen | Biological Psychiatry | 13 / 13 | 43 / 42 | 15050867 | **1** |  |  |  |  |  |  |  |  |  |  |  |  |  |  |  |  |  |  |  |  |
| 2003 | Callicott | American Journal of Psychiatry | 14 / 14 | 32 / 33 | 14638592 | **1** |  |  |  |  |  |  |  |  |  |  |  |  |  |  |  |  |  |  |  |  |
| 2003 | Honey | Psychological Medicine | 30 / 27 | 37 / 35 | 12946085 | **1** |  |  |  |  |  |  |  |  |  |  |  |  |  |  |  |  |  |  |  |  |
| 2003 | Quintana | Biological Psychiatry | 8 / 8 | 35 / 29 | 12814861 | **1** |  |  |  |  |  |  |  |  |  |  |  |  |  |  |  |  |  |  |  |  |
| 2003 | Sabri | Journal of Nuclear Medicine | 11 / 10 | 31 / 31 | 12732667 | **1** |  |  |  |  |  |  |  |  |  |  |  |  |  |  |  |  |  |  |  |  |
| 2003 | Walter | Schizophrenia Research | 15 / 15 | 29 / 30 | 12729869 | **1** |  |  |  |  |  |  |  |  |  |  |  |  |  |  |  |  |  |  |  |  |
| 2003 | Perlstein | Biological Psychiatry | 16 / 15 | 37 / 36 | 12513942 | **1** |  |  |  |  |  |  |  |  |  |  |  |  |  |  |  |  |  |  |  |  |
| 2002 | Barch | Journal of Abnormal Psychology | 38 / 48 | 36 / 37 | 12150424 | **1** |  |  |  |  |  |  |  |  |  |  |  |  |  |  |  |  |  |  |  |  |
| 2002 | Wykes | British Journal of Psychiatry | 12 / 6 | 36 / 36 | 12151286 | **1** |  |  |  |  |  |  |  |  |  |  |  |  |  |  |  |  |  |  |  |  |
| 2002 | Honey | Schizophrenia Research | 20 / 20 | 35 / 39 | 11728837 | **1** |  |  |  |  |  |  |  |  |  |  |  |  |  |  |  |  |  |  |  |  |
| 2001 | Meyer-Lindenberg | American Journal of Psychiatry | 13 / 13 | 33 / 30 | 11691686 | **1** |  |  |  |  |  |  |  |  |  |  |  |  |  |  |  |  |  |  |  |  |
| 2001 | Perlstein | American Journal of Psychiatry | 17 / 16 | 37 / 37 | 11431233 | **1** |  |  |  |  |  |  |  |  |  |  |  |  |  |  |  |  |  |  |  |  |
| 2000 | Callicott | Cerebral Cortex | 13 / 18 | 34 / 30 | 11053229 | **1** |  |  |  |  |  |  |  |  |  |  |  |  |  |  |  |  |  |  |  |  |
| 2012 | Stolz | Neuroimage | 22 / 28 | 28 / 27 | 22992490 |  | **1** |  |  |  |  |  |  |  |  |  |  |  |  |  |  |  |  |  |  |  |
| 2012 | Hutcheson | Schizophrenia Research | 28 / 28 | 37 / 36 | 22831772 |  | **1** |  |  |  |  |  |  |  |  |  |  |  |  |  |  |  |  |  |  |  |
| 2013 | Choi | European Archives of Psychiatry and Clinical Neuroscience | 14 / 15 | 29 / 28 | 22678652 |  | **1** |  |  |  |  |  |  |  |  |  |  |  |  |  |  |  |  |  |  |  |
| 2012 | Cuervo-Lombard | British Journal of Psychiatry | 13 / 14 | 31 / 30 | 22539776 |  | **1** |  |  |  |  |  |  |  |  |  |  |  |  |  |  |  |  |  |  |  |
| 2011 | Wolf | Frontiers in Behavioral Neuroscience | 26 / 25 | 38 / 38 | 22355285 |  | **1** |  |  |  |  |  |  |  |  |  |  |  |  |  |  |  |  |  |  |  |
| 2010 | Zierhurt | Psychiatry Research: Neuroimaging | 11 / 13 | 29 / 25 | 20702070 |  | **1** |  |  |  |  |  |  |  |  |  |  |  |  |  |  |  |  |  |  |  |
| 2010 | Lepage | Psychiatry Research: Neuroimaging | 15 / 18 | 34 / 29 | 20488673 |  | **1** |  |  |  |  |  |  |  |  |  |  |  |  |  |  |  |  |  |  |  |
| 2010 | Hall | Psychological Medicine | 15 / 14 | 38 / 34 | 19732478 |  | **1** |  |  |  |  |  |  |  |  |  |  |  |  |  |  |  |  |  |  |  |
| 2009 | Rametti | European Archives of Psychiatry and Clinical Neuroscience | 22 / 24 | 32 / 32 | 19224116 |  | **1** |  |  |  |  |  |  |  |  |  |  |  |  |  |  |  |  |  |  |  |
| 2010 | Sergerie | Schizophrenia Bulletin | 20 / 20 | 32 / 29 | 19176471 |  | **1** |  |  |  |  |  |  |  |  |  |  |  |  | **1** |  |  |  |  |  |  |
| 2008 | Bonner-Jackson | Psychiatry Research: Neuroimaging | 18 / 15 | 40 / 43 | 18790618 |  | **1** |  |  |  |  |  |  |  |  |  |  |  |  |  |  |  |  |  |  |  |
| 2007 | Achim | Archives of General Psychiatry | 26 / 20 | 23 / 24 | 17768265 |  | **1** |  |  |  |  |  |  |  |  |  |  |  |  |  |  |  |  |  |  |  |
| 2006 | Weiss | Biological Psychiatry | 16 / 16 | 47 / 47 | 17020747 |  | **1** |  |  |  |  |  |  |  |  |  |  |  |  |  |  |  |  |  |  |  |
| 2006 | Lepage | Biological Psychiatry | 15 / 18 | 34 / 29 | 16814264 |  | **1** |  |  |  |  |  |  |  |  |  |  |  |  |  |  |  |  |  |  |  |
| 2006 | Ongur | Archives of General Psychiatry | 15 / 15 | 40 / 38 | 16585464 |  | **1** |  |  |  |  |  |  |  |  |  |  |  |  |  |  |  |  |  |  |  |
| 2005 | Ragland | American Journal of Psychiatry | 14 / 14 | 35 / 31 | 16199830 |  | **1** |  |  |  |  |  |  |  |  |  |  |  |  |  |  |  |  |  |  |  |
| 2006 | Assaf | Biological Psychiatry | 16 / 16 | 39 / 38 | 16199012 |  | **1** |  |  |  |  |  |  |  |  |  |  |  |  |  |  |  |  |  |  |  |
| 2005 | Bonner-Jackson | Biological Psychiatry | 17 / 26 | 22 / 21 | 15992522 |  | **1** |  |  |  |  |  |  |  |  |  |  |  |  |  |  |  |  |  |  |  |
| 2004 | Ragland | American Journal of Psychiatry | 14 / 15 | 33 / 28 | 15169688 |  | **1** |  |  |  |  |  |  |  |  |  |  |  |  |  |  |  |  |  |  |  |
| 2003 | Hofer | American Journal of Psychiatry | 10 / 10 | 31 / 29 | 14514494 |  | **1** |  |  |  |  |  |  |  |  |  |  |  |  |  |  |  |  |  |  |  |
| 2003 | Leube | Schizophrenia Research | 10 / 9 | NR / NR | 14511805 |  | **1** |  |  |  |  |  |  |  |  |  |  |  |  |  |  |  |  |  |  |  |
| 2003 | Jessen | American Journal of Psychiatry | 12 / 12 | 27 / 28 | 12832246 |  | **1** |  |  |  |  |  |  |  |  |  |  |  |  |  |  |  |  |  |  |  |
| 2003 | Hofer | American Journal of Psychiatry | 10 / 10 | 33 / 29 | 12727695 |  | **1** |  |  |  |  |  |  |  |  |  |  |  |  |  |  |  |  |  |  |  |
| 2003 | Eyler Zorrilla | Schizophrenia Research | 9 / 10 | 55 / 62 | 12414075 |  | **1** |  |  |  |  |  |  |  |  |  |  |  |  |  |  |  |  |  |  |  |
| 2001 | Ragland | American Journal of Psychiatry | 23 / 23 | 35 / 31 | 11431234 |  | **1** |  |  |  |  |  |  |  |  |  |  |  |  |  |  |  |  |  |  |  |
| 2001 | Crespo-Facorro | Human Brain Mapping | 19 / 34 | 30 / 26 | 11241873 |  | **1** |  |  |  |  |  |  |  |  |  |  |  |  |  |  |  |  |  |  |  |
| 2000 | Heckers | Biological Psychiatry | 9 / 8 | 41 / 43 | 11032976 |  | **1** |  |  |  |  |  |  |  |  |  |  |  |  |  |  |  |  |  |  |  |
| 1999 | Heckers | Archives of General Psychiatry | 16 / 8 | 41 / 40 | 10591289 |  | **1** |  |  |  |  |  |  |  |  |  |  |  |  |  |  |  |  |  |  |  |
| 1998 | Heckers | Nature Neuroscience | 13 / 8 | 42 / 40 | 10195166 |  | **1** |  |  |  |  |  |  |  |  |  |  |  |  |  |  |  |  |  |  |  |
| 1999 | Crespo-Facorro | American Journal of Psychiatry | 14 / 13 | 31 / 29 | 10080553 |  | **1** |  |  |  |  |  |  |  |  |  |  |  |  |  |  |  |  |  |  |  |
| 1998 | Fletcher | Archives of General Psychiatry | 12 / 7 | NR / NR | 9819069 |  | **1** |  |  |  |  |  |  |  |  |  |  |  |  |  |  |  |  |  |  |  |
| 1998 | Wiser | Neuroreport | 15 / 29 | NR / NR | 9665622 |  | **1** |  |  |  |  |  |  |  |  |  |  |  |  |  |  |  |  |  |  |  |
| 1997 | Ganguli | Biological Psychiatry | 4 / 4 | 40 / 37 | 8988793 |  | **1** |  |  |  |  |  |  |  |  |  |  |  |  |  |  |  |  |  |  |  |
| 1996 | Andreasen | Proceedings of the National Academy of Sciences U S A | 14 / 13 | 31 / 29 | 8790444 |  | **1** |  |  |  |  |  |  |  |  |  |  |  |  |  |  |  |  |  |  |  |
| 2011 | Smee | Acta Psychiatrica Scandinavica | 9 / 9 | 37 / 33 | 21083650 |  |  | **1** |  |  |  |  |  |  |  |  |  |  |  |  |  |  |  |  |  |  |
| 2009 | Costafreda | Human Brain Mapping | 39 / 48 | 36 / 37 | 19479729 |  |  | **1** |  |  |  |  |  |  |  |  |  |  |  |  |  |  |  |  |  |  |
| 2008 | Kircher | Schizophrenia Research | 12 / 12 | 27 / 27 | 18356025 |  |  | **1** |  |  |  |  |  |  |  |  |  |  |  |  |  |  |  |  |  |  |
| 2008 | Ragland | Schizophrenia Research | 13 / 14 | 36 / 34 | 18155880 |  |  | **1** |  |  |  |  |  |  |  |  |  |  |  |  |  |  |  |  |  |  |
| 2005 | Boksman | Schizophrenia Research | 10 / 10 | 23 / 22 | 15885517 |  |  | **1** |  |  |  |  |  |  |  |  |  |  |  |  |  |  |  |  |  |  |
| 2005 | Fu | American Journal of Psychiatry | 19 / 11 | 35 / 30 | 15741465 |  |  | **1** |  |  |  |  |  |  |  |  |  |  |  |  |  |  |  |  |  |  |
| 2004 | Jones | Biological Psychiatry | 14 / 8 | 28 / 27 | 15601603 |  |  | **1** |  |  |  |  |  |  |  |  |  |  |  |  |  |  |  |  |  |  |
| 2003 | Shergill | British Journal of Psychiatry | 8 / 8 | 31 / 29 | 12777344 |  |  | **1** |  |  |  |  |  |  |  |  |  |  |  |  |  |  |  |  |  |  |
| 2002 | Kircher | Psychological Medicine | 6 / 6 | 34 / 34 | 11989989 |  |  | **1** |  |  |  |  |  |  |  |  |  |  |  |  |  |  |  |  |  |  |
| 2000 | Shergill | American Journal of Psychiatry | 8 / 6 | 32 / 34 | 11007729 |  |  | **1** |  |  |  |  |  |  |  |  |  |  |  |  |  |  |  |  |  |  |
| 1999 | Curtis | Schizophrenia Research | 5 / 5 | 30 / 32 | 10227106 |  |  | **1** | **1** |  |  |  |  |  |  |  |  |  |  |  |  |  |  |  |  |  |
| 1996 | Fletcher | The Journal of Neuroscience | 12 / 12 | 26 / NR | 8824341 |  |  | **1** |  |  |  |  |  |  |  |  |  |  |  |  |  |  |  |  |  |  |
| 1996 | McGuire | British Journal of Psychiatry | 12 / 6 | 33 / 28 | 8871790 |  |  | **1** |  |  |  |  |  |  |  |  |  |  |  |  |  |  |  |  |  |  |
| 2013 | Chen | Neuroreport | 20 / 20 | 29 / 31 | 23324649 |  |  |  | **1** |  |  |  |  |  |  |  |  |  |  |  |  |  |  |  |  |  |
| 2013 | Jamadar | Biological Psychiatry | 74 / 133 | 36 / 32 | 22985694 |  |  |  | **1** |  |  |  |  |  |  |  |  |  |  |  |  |  |  |  |  |  |
| 2012 | Arcuri | Schizophrenia Research and Treatment | 18 / 10 | 35 / 35 | 22966432 |  |  |  | **1** |  |  |  |  |  |  |  |  |  |  |  |  |  |  |  |  |  |
| 2012 | Straube | Human Brain Mapping | 16 / 16 | 38 / 28 | 22378493 |  |  |  | **1** |  |  |  |  |  |  |  |  |  |  |  |  |  |  |  |  |  |
| 2010 | Borofsky | Journal of Neurolinguistics | 14 / 14 | 13 / 12 | 22147958 |  |  |  | **1** |  |  |  |  |  |  |  |  |  |  |  |  |  |  |  |  |  |
| 2009 | Kim | Journal of the Int Neuropsychological Society | 12 / 12 | 25 / 25 | 19709452 |  |  |  | **1** |  |  |  |  |  |  |  |  |  |  |  |  |  |  |  |  |  |
| 2008 | Griego | Brain and Language | 8 / 10 | 32 / 32 | 18829095 |  |  |  | **1** |  |  |  |  |  |  |  |  |  |  |  |  |  |  |  |  |  |
| 2008 | Dollfus | Schizophrenia Research | 23 / 23 | 30 / 30 | 18178386 |  |  |  | **1** |  |  |  |  |  |  |  |  |  |  |  |  |  |  |  |  |  |
| 2007 | Kuperberg | Archives of General Psychiatry | 17 / 15 | 45 / 42 | 17283282 |  |  |  | **1** |  |  |  |  |  |  |  |  |  |  |  |  |  |  |  |  |  |
| 2007 | Han | Neuroimage | 12 / 12 | 40 / 46 | 17215145 |  |  |  | **1** |  |  |  |  |  |  |  |  |  |  |  |  |  |  |  |  |  |
| 2006 | Stephane | Journal of Psychiatry and Neuroscience | 18 / 12 | 37 / 42 | 17136217 |  |  |  | **1** |  |  |  |  |  |  |  |  |  |  |  |  |  |  |  |  |  |
| 2007 | Kircher | Neuroimage | 12 / 12 | 33 / 29 | 17081771 |  |  |  | **1** |  |  |  |  |  |  |  |  |  |  |  |  |  |  |  |  |  |
| 2006 | Koeda | Biological Psychiatry | 14 / 14 | 32 / 29 | 16616721 |  |  |  | **1** |  |  |  |  |  |  |  |  |  |  |  |  |  |  |  |  |  |
| 2005 | Kircher | British Journal of Psychiatry | 6 / 6 | 34 / 34 | 15738501 |  |  |  | **1** |  |  |  |  |  |  |  |  |  |  |  |  |  |  |  |  |  |
| 2004 | Tendolkar | Neuroscience Letters | 12 / 12 | NR / NR | 15380297 |  |  |  | **1** |  |  |  |  |  |  |  |  |  |  |  |  |  |  |  |  |  |
| 2003 | Kubicki | Neuroimage | 9 / 9 | 40 / 43 | 14683698 |  |  |  | **1** |  |  |  |  |  |  |  |  |  |  |  |  |  |  |  |  |  |
| 2001 | Kircher | Schizophrenia Research | 12 / 7 | 33 / 34 | 11378312 |  |  |  | **1** |  |  |  |  |  |  |  |  |  |  |  |  |  |  |  |  |  |
| 2001 | Surguladze | Psychiatry Research: Neuroimaging | 14 / 7 | 36 / 36 | 11231095 |  |  |  | **1** |  |  |  |  |  |  |  |  |  |  |  |  |  |  |  |  |  |
| 2013 | Smucny | PLoS One | 21 / 23 | 47 / 39 | 23560100 |  |  |  |  | **1** |  |  |  |  |  |  |  |  |  |  |  |  |  |  |  |  |
| 2013 | Sambataro | Neuropsychopharmacology | 70 / 235 | 32 / 32 | 23299932 |  |  |  |  | **1** |  |  |  |  |  |  |  |  |  |  |  |  |  |  |  |  |
| 2012 | Tregellas | Schizophrenia Research | 22 / 17 | 38 / 35 | 23062751 |  |  |  |  | **1** |  |  |  |  |  |  |  |  |  |  |  |  |  |  |  |  |
| 2011 | Becerril | Neuroimage | 37 / 32 | 37 / 36 | 20883800 |  |  |  |  | **1** |  |  |  |  |  |  |  |  |  |  |  |  |  |  |  |  |
| 2011 | Hasenkamp | Schizophrenia Research | 10 / 10 | 43 / 39 | 20869846 |  |  |  |  | **1** |  |  |  |  |  |  |  |  |  |  |  |  |  |  |  |  |
| 2010 | Silverstein | Neuropsychologia | 14 / 16 | 33 / 29 | 20678981 |  |  |  |  | **1** |  |  |  |  |  |  |  |  |  |  |  |  |  |  |  |  |
| 2010 | Edwards | Frontiers in Human Neuroscience | 22 / 14 | 35 / 36 | 20461148 |  |  |  |  | **1** |  |  |  |  |  |  |  |  |  |  |  |  |  |  |  |  |
| 2010 | McAllindon | Schizophrenia Research | 15 / 14 | 37 / 36 | 20153139 |  |  |  |  | **1** |  |  |  |  |  |  |  |  |  |  |  |  |  |  |  |  |
| 2011 | Hong | Schizophrenia Bulletin | 20 / 24 | 36 / 35 | 19713300 |  |  |  |  | **1** |  |  |  |  |  |  |  |  |  |  |  |  |  |  |  |  |
| 2010 | Blasi | Cerebral Cortex | 16 / 21 | 33 / 29 | 19633177 |  |  |  |  | **1** |  |  |  |  |  |  |  |  |  |  |  |  |  |  |  |  |
| 2009 | Keedy | Psychiatry Research: Neuroimaging | 9 / 9 | NR / NR | 19243925 |  |  |  |  | **1** |  |  |  |  |  |  |  |  |  |  |  |  |  |  |  |  |
| 2009 | Woodward | Schizophrenia Research | 25 / 32 | 27 / 27 | 19179050 |  |  |  |  | **1** |  |  |  |  |  |  |  |  |  |  |  |  |  |  |  |  |
| 2009 | Schirmer | Progress in Neuro-Psychopharmacology and Biol. Psych. | 10 / 20 | 35 / 29 | 18957312 |  |  |  |  | **1** |  |  |  |  |  |  |  |  |  |  |  |  |  |  |  |  |
| 2009 | Stern | Human Brain Mapping | 17 / 21 | 39 / 40 | 18819107 |  |  |  |  | **1** |  |  |  |  |  |  |  |  |  |  |  |  |  |  |  |  |
| 2008 | Yoon | American Journal of Psychiatry | 25 / 24 | 20 / 22 | 18519527 |  |  |  |  | **1** |  |  |  |  |  |  |  |  |  |  |  |  |  |  |  |  |
| 2008 | Wolf | Brain Imaging and Behavior | 17 / 21 | 32 / 29 | 19756228 |  |  |  |  | **1** |  |  |  |  |  |  |  |  |  |  |  |  |  |  |  |  |
| 2006 | Liddle | Psychological Medicine | 28 / 28 | 32 / 28 | 16650349 |  |  |  |  | **1** |  |  |  |  |  |  |  |  |  |  |  |  |  |  |  |  |
| 2006 | Calhoun | Human Brain Mapping | 15 / 15 | 37 / 38 | 16108017 |  |  |  |  | **1** |  |  |  |  |  |  |  |  |  |  |  |  |  |  |  |  |
| 2005 | Holmes | Schizophrenia Research | 7 / 9 | 39 / 34 | 15949653 |  |  |  |  | **1** |  |  |  |  |  |  |  |  |  |  |  |  |  |  |  |  |
| 2005 | Laurens | Schizophrenia Research | 28 / 28 | 32 / 28 | 15885507 |  |  |  |  | **1** |  |  |  |  |  |  |  |  |  |  |  |  |  |  |  |  |
| 2005 | Kiehl | Biological Psychiatry | 18 / 18 | 35 / 36 | 15860344 |  |  |  |  | **1** |  |  |  |  |  |  |  |  |  |  |  |  |  |  |  |  |
| 2005 | MacDonald | Journal of Abnormal Psychology | 18 / 28 | 28 / 25 | 15741464 |  |  |  |  | **1** |  |  |  |  |  |  |  |  |  |  |  |  |  |  |  |  |
| 2004 | Eyler | Psychiatry Research: Neuroimaging | 9 / 10 | 59 / 60 | 15135158 |  |  |  |  | **1** |  |  |  |  |  |  |  |  |  |  |  |  |  |  |  |  |
| 2004 | Salgado-Pineda | Neuroimage | 14 / 14 | 25 / 25 | 15006650 |  |  |  |  | **1** |  |  |  |  |  |  |  |  |  |  |  |  |  |  |  |  |
| 2004 | Hugdahl | American Journal of Psychiatry | 12 / 12 | 32 / 31 | 14754778 |  |  |  |  | **1** |  |  |  |  |  |  |  |  |  |  |  |  |  |  |  |  |
| 2003 | MacDonald | Journal of Abnormal Psychology | 17 / 17 | 34 / 34 | 14674880 |  |  |  |  | **1** |  |  |  |  |  |  |  |  |  |  |  |  |  |  |  |  |
| 2003 | Ngan | Neuroimage | 14 / 29 | 35 / 29 | 14568459 |  |  |  |  | **1** |  |  |  |  |  |  |  |  |  |  |  |  |  |  |  |  |
| 2002 | Ojeda | Human Brain Mapping | 11 / 10 | 28 / 26 | 12353245 |  |  |  |  | **1** |  |  |  |  |  |  |  |  |  |  |  |  |  |  |  |  |
| 2001 | Carter | American Journal of Psychiatry | 17 / 16 | 34 / 34 | 11532726 |  |  |  |  | **1** |  |  |  |  |  |  |  |  |  |  |  |  |  |  |  |  |
| 2001 | Kiehl | Schizophrenia Research | 11 / 11 | 27 / 27 | 11295369 |  |  |  |  | **1** |  |  |  |  |  |  |  |  |  |  |  |  |  |  |  |  |
| 2001 | Barch | Archives of General Psychiatry | 14 / 12 | 24 / 25 | 11231835 |  |  |  |  | **1** |  |  |  |  |  |  |  |  |  |  |  |  |  |  |  |  |
| 2012 | Vercammen | Journal of Psychiatry and Neuroscience | 20 / 23 | 34 / 33 | 22617625 |  |  |  |  |  | **1** |  |  |  |  |  |  |  |  | **1** |  |  |  |  |  |  |
| 2012 | Hughes | Biological Psychology | 10 / 10 | 36 / 35 | 22027085 |  |  |  |  |  | **1** |  |  |  |  |  |  |  |  |  |  |  |  |  |  |  |
| 2011 | Zandbelt | Biological Psychiatry | 24 / 24 | 31 / 32 | 21903198 |  |  |  |  |  | **1** |  |  |  |  |  |  |  |  |  |  |  |  |  |  |  |
| 2010 | Jamadar | Neuropsychologia | 11 / 11 | 37 / 37 | 20036266 |  |  |  |  |  | **1** |  |  |  |  |  |  |  |  |  |  |  |  |  |  |  |
| 2010 | Ungar | Psychiatry Research: Neuroimaging | 15 / 15 | 43 / 43 | 19963356 |  |  |  |  |  | **1** |  |  |  |  |  |  |  |  |  |  |  |  |  |  |  |
| 2008 | Barkataki | Behavioral Sciences and the Law | 24 / 14 | 35 / 32 | 18327832 |  |  |  |  |  | **1** |  |  |  |  |  |  |  |  |  |  |  |  |  |  |  |
| 2008 | Choi | Psychiatry Investigation | 10 / 10 | 29 / 30 | 20046409 |  |  |  |  |  | **1** |  |  |  |  |  |  |  |  |  |  |  |  |  |  |  |
| 2007 | Kaladjian | Schizophrenia Research | 21 / 21 | 35 / 36 | 17855057 |  |  |  |  |  | **1** |  |  |  |  |  |  |  |  |  |  |  |  |  |  |  |
| 2007 | Harrison | Acta Psychiatrica Scandinavica | 12 / 14 | 32 / 32 | 17803757 |  |  |  |  |  | **1** |  |  |  |  |  |  |  |  |  |  |  |  |  |  |  |
| 2007 | Joyal | Schizophrenia Research | 36 / 12 | NR / NR | 17291724 |  |  |  |  |  | **1** |  |  |  |  |  |  |  |  |  |  |  |  |  |  |  |
| 2007 | Yucel | Acta Psychiatrica Scandinavica | 8 / 8 | 21 / 22 | 17244179 |  |  |  |  |  | **1** |  |  |  |  |  |  |  |  |  |  |  |  |  |  |  |
| 2007 | Weiss | Psychiatry Research: Neuroimaging | 8 / 8 | 30 / 27 | 17188464 |  |  |  |  |  | **1** |  |  |  |  |  |  |  |  |  |  |  |  |  |  |  |
| 2006 | Arce | Neuroimage | 17 / 17 | 41 / 40 | 16766210 |  |  |  |  |  | **1** |  |  |  |  |  |  |  |  |  |  |  |  |  |  |  |
| 2005 | Kerns | American Journal of Psychiatry | 13 / 13 | 36 / 36 | 16199829 |  |  |  |  |  | **1** |  |  |  |  |  |  |  |  |  |  |  |  |  |  |  |
| 2005 | Jeong | Psychiatry Research: Neuroimaging | 10 / 10 | 29 / 30 | 16054343 |  |  |  |  |  | **1** |  |  |  |  |  |  |  |  |  |  |  |  |  |  |  |
| 2003 | Weiss | Psychiatry Research | 13 / 13 | 33 / 30 | 12738340 |  |  |  |  |  | **1** |  |  |  |  |  |  |  |  |  |  |  |  |  |  |  |
| 2003 | Laurens | Brain | 16 / 10 | 33 / 32 | 12566282 |  |  |  |  |  | **1** |  |  |  |  |  |  |  |  |  |  |  |  |  |  |  |
| 2003 | Weiss | Biological Psychiatry | 12 / 12 | 48 / 49 | 12513944 |  |  |  |  |  | **1** |  |  |  |  |  |  |  |  |  |  |  |  |  |  |  |
| 2002 | Erkwoh | Psychiatry Research: Neuroimaging | 20 / 10 | 34 / 24 | 12208491 |  |  |  |  |  | **1** |  |  |  |  |  |  |  |  |  |  |  |  |  |  |  |
| 2001 | Rubia | Schizophrenia Research | 6 / 7 | 40 / 40 | 11595391 |  |  |  |  |  | **1** |  |  |  |  |  |  |  |  |  |  |  |  |  |  |  |
| 1997 | Carter | American Journal of Psychiatry | 14 / 15 | 36 / 34 | 9396944 |  |  |  |  |  | **1** |  |  |  |  |  |  |  |  |  |  |  |  |  |  |  |
| 2012 | Dowd | PLoS One | 25 / 20 | 31 / 33 | 22574121 |  |  |  |  |  |  | **1** |  |  |  |  |  |  |  |  |  |  |  | **1** |  |  |
| 2012 | Pedersen | Brain and Cognition | 36 / 25 | 28 / 31 | 22554566 |  |  |  |  |  |  | **1** |  |  |  |  |  |  |  |  |  |  |  |  |  |  |
| 2010 | Wilmsmeier | Journal of Psychiatry and Neuroscience | 36 / 28 | 28 / 31 | 20731964 |  |  |  |  |  |  | **1** |  |  |  |  |  |  |  |  |  |  |  |  |  |  |
| 2010 | Rowland | Schizophrenia Bulletin | 17 / 17 | 42 / 41 | 20418447 |  |  |  |  |  |  | **1** |  |  |  |  |  |  |  |  |  |  |  |  |  |  |
| 2010 | Koch | Neuroimage | 19 / 20 | 35 / 30 | 20006717 |  |  |  |  |  |  | **1** |  |  |  |  |  |  |  |  |  |  |  | **1** |  |  |
| 2009 | Weickert | The Journal of Neuroscience | 31 / 25 | 30 / 29 | 19176832 |  |  |  |  |  |  | **1** |  |  |  |  |  |  |  |  |  |  |  |  |  |  |
| 2008 | Eyler | Psychiatry Research: Neuroimaging | 17 / 14 | 47 / 46 | 18055184 |  |  |  |  |  |  | **1** |  |  |  |  |  |  |  |  |  |  |  |  |  |  |
| 2008 | Jensen | Neuropsychopharmacology | 13 / 13 | 38 / 37 | 17473838 |  |  |  |  |  |  | **1** |  |  |  |  |  |  |  |  |  |  |  |  |  |  |
| 2007 | Koch | Neuroscience | 13 / 13 | 26 / 27 | 17448605 |  |  |  |  |  |  | **1** |  |  |  |  |  |  |  |  |  |  |  |  |  |  |
| 2006 | Zedkova | Schizophrenia Research | 10 / 15 | 34 / 31 | 16945506 |  |  |  |  |  |  | **1** |  |  |  |  |  |  |  |  |  |  |  |  |  |  |
| 2006 | Reiss | Schizophrenia Research | 10 / 10 | 29 / 26 | 16814986 |  |  |  |  |  |  | **1** |  |  |  |  |  |  |  |  |  |  |  |  |  |  |
| 2006 | Heinze | Schizophrenia Research | 18 / 15 | 36 / 31 | 16497485 |  |  |  |  |  |  | **1** |  |  |  |  |  |  |  |  |  |  |  |  |  |  |
| 2010 | Simons | Biological Psychiatry | 15 / 12 | 35 / 34 | 19846064 |  |  |  |  |  |  |  | **1** |  |  |  |  |  |  |  |  |  |  |  |  |  |
| 2009 | Szycik | Schizophrenia Research | 15 / 15 | 38 / 37 | 19303257 |  |  |  |  |  |  |  | **1** | **1** |  |  |  |  |  |  |  |  |  |  |  |  |
| 2008 | Morey | Journal of Neuropsychiatry and Clinical Neuroscience | 26 / 17 | 30 / 28 | 19196926 |  |  |  |  |  |  |  | **1** |  |  |  |  |  |  |  |  |  |  |  |  |  |
| 2009 | Tregellas | American Journal of Psychiatry | 18 / 17 | 37 / 37 | 19147695 |  |  |  |  |  |  |  | **1** |  |  |  |  |  |  |  |  |  |  |  |  |  |
| 2009 | Kang | Schizophrenia Research | 28 / 28 | 30 / 30 | 18818053 |  |  |  |  |  |  |  | **1** |  |  |  |  |  |  | **1** |  |  |  |  |  |  |
| 2008 | Zhang | Acta Psychiatrica Scandinavica | 26 / 13 | 31 / 32 | 18759811 |  |  |  |  |  |  |  | **1** |  |  |  |  |  |  |  |  |  |  |  |  |  |
| 2008 | Zhang | Journal of Psychiatric Research | 26 / 13 | 31 / 32 | 17673231 |  |  |  |  |  |  |  | **1** |  |  |  |  |  |  |  |  |  |  |  |  |  |
| 2003 | Copolov | Psychiatry Research: Neuroimaging | 15 / 8 | 32 / 32 | 12694889 |  |  |  |  |  |  |  | **1** |  |  |  |  |  |  |  |  |  |  |  |  |  |
| 2002 | Braus | Archives of General Psychiatry | 12 / 11 | 25 / 29 | 12150645 |  |  |  |  |  |  |  | **1** |  |  |  |  |  |  |  |  |  |  |  |  |  |
| 2000 | Holcomb | American Journal of Psychiatry | 18 / 12 | 31 / 28 | 11007718 |  |  |  |  |  |  |  | **1** |  |  |  |  |  |  |  |  |  |  |  |  |  |
| 2010 | Silverstein | Psychological Medicine | 14 / 13 | 33 / 30 | 19895721 |  |  |  |  |  |  |  |  | **1** |  |  |  |  |  |  |  |  |  |  |  |  |
| 2009 | Silverstein | Journal of Integrative Neuroscience | 14 / 14 | 33 / 30 | 19618486 |  |  |  |  |  |  |  |  | **1** |  |  |  |  |  |  |  |  |  |  |  |  |
| 2010 | Takahashi | Schizophrenia Bulletin | 12 / 12 | 32 / 29 | 18927345 |  |  |  |  |  |  |  |  | **1** |  |  |  |  |  |  |  |  |  |  |  |  |
| 2008 | Martinez | The Journal of Neuroscience | 13 / 11 | 36 / 31 | 18650327 |  |  |  |  |  |  |  |  | **1** |  |  |  |  |  |  |  |  |  |  |  |  |
| 2002 | Heckers | Psychiatry Research: Neuroimaging | 11 / 10 | 48 / 48 | 12426031 |  |  |  |  |  |  |  |  | **1** |  |  |  |  |  |  |  |  |  |  |  |  |
| 2006 | Plailly | Neuroimage | 12 / 12 | 30 / 33 | 16099179 |  |  |  |  |  |  |  |  |  | **1** |  |  |  |  |  |  |  |  |  |  |  |
| 2001 | Crespo-Facorro | Journal of the American Medical Association (JAMA) | 18 / 16 | 30 / 30 | 11466121 |  |  |  |  |  |  |  |  |  | **1** |  |  |  |  | **1** |  |  |  |  |  |  |
| 2013 | Ledoux | Psychiatry Research: Neuroimaging | 21 / 22 | 32 / 30 | 23352276 |  |  |  |  |  |  |  |  |  |  | **1** |  |  |  |  |  |  |  |  |  |  |
| 2010 | Jimenez | Schizophrenia Research | 33 / 35 | 33 / 32 | 20385471 |  |  |  |  |  |  |  |  |  |  | **1** |  |  |  |  |  |  |  |  |  |  |
| 2011 | Davalos | Schizophrenia Research | 16 / 18 | 48 / 41 | 20674279 |  |  |  |  |  |  |  |  |  |  |  | **1** |  |  |  |  |  |  |  |  |  |
| 2005 | Ortuño | Neuroimage | 11 / 10 | 28 / 26 | 15627600 |  |  |  |  |  |  |  |  |  |  |  | **1** |  |  |  |  |  |  |  |  |  |
| 2012 | Minzenberg | Psychiatry Research: Neuroimaging | 27 / 18 | 27 / 34 | 22608155 |  |  |  |  |  |  |  |  |  |  |  |  | **1** |  |  |  |  |  |  |  |  |
| 2009 | Scheuerecker | Journal of Psychiatric Research | 12 / 12 | 36 / 36 | 18951556 |  |  |  |  |  |  |  |  |  |  |  |  | **1** |  |  |  |  |  |  |  |  |
| 2008 | Hazlett | Neuroimage | 13 / 13 | 39 / 36 | 18588988 |  |  |  |  |  |  |  |  |  |  |  |  | **1** |  |  |  |  |  |  |  |  |
| 2008 | Rowland | Psychiatry Research: Neuroimaging | 8 / 8 | 36 / 22 | 18407471 |  |  |  |  |  |  |  |  |  |  |  |  | **1** |  |  |  |  |  |  |  |  |
| 2004 | Bertolino | Biological Psychiatry | 17 / 17 | 26 / 26 | 15271588 |  |  |  |  |  |  |  |  |  |  |  |  | **1** |  |  |  |  |  |  |  |  |
| 2004 | Payoux | Movement Disorders | 6 / 6 | 36 / 38 | 14743365 |  |  |  |  |  |  |  |  |  |  |  |  | **1** |  |  |  |  |  |  |  |  |
| 1998 | Spence | British Journal of Psychiatry | 13 / 6 | 37 / 31 | 9715333 |  |  |  |  |  |  |  |  |  |  |  |  | **1** |  |  |  |  |  |  |  |  |
| 1997 | Spence | Brain | 13 / 6 | 37 / NR | 9397017 |  |  |  |  |  |  |  |  |  |  |  |  | **1** |  |  |  |  |  |  |  |  |
| 2012 | Nagel | PLoS One | 17 / 16 | 30 / 28 | 22693639 |  |  |  |  |  |  |  |  |  |  |  |  |  | **1** |  |  |  |  |  |  |  |
| 2008 | Polli | Brain | 18 / 15 | 42 / 37 | 18158315 |  |  |  |  |  |  |  |  |  |  |  |  |  | **1** |  |  |  |  |  |  |  |
| 2007 | Nagel | Neuroimage | 17 / 16 | 36 / 34 | 17011791 |  |  |  |  |  |  |  |  |  |  |  |  |  | **1** |  |  |  |  |  |  |  |
| 2006 | Keedy | Psychiatry Research: Neuroimaging | 15 / 24 | 25 / 25 | 16571373 |  |  |  |  |  |  |  |  |  |  |  |  |  | **1** |  |  |  |  |  |  |  |
| 2005 | Hong | Biological Psychiatry | 12 / 12 | 40 / 38 | 15820229 |  |  |  |  |  |  |  |  |  |  |  |  |  | **1** |  |  |  |  |  |  |  |
| 2005 | Lencer | Neuroimage | 17 / 16 | 36 / 34 | 15670704 |  |  |  |  |  |  |  |  |  |  |  |  |  | **1** |  |  |  |  |  |  |  |
| 2004 | Tregellas | American Journal of Psychiatry | 14 / 14 | 40 / 38 | 14754781 |  |  |  |  |  |  |  |  |  |  |  |  |  | **1** |  |  |  |  |  |  |  |
| 2002 | Raemaekers | Archives of General Psychiatry | 16 / 17 | 28 / 26 | 11926931 |  |  |  |  |  |  |  |  |  |  |  |  |  | **1** |  |  |  |  |  |  |  |
| 2002 | McDowell | Biological Psychiatry | 14 / 13 | 37 / 35 | 11839364 |  |  |  |  |  |  |  |  |  |  |  |  |  | **1** |  |  |  |  |  |  |  |
| 2013 | Mendrek | ISRN Psychiatry | 21 / 23 | 29 / 33 | 23738207 |  |  |  |  |  |  |  |  |  |  |  |  |  |  | **1** |  |  |  |  |  |  |
| 2013 | Linnman | Schizophrenia Research | 15 / 13 | 32 / 36 | 23301307 |  |  |  |  |  |  |  |  |  |  |  |  |  |  | **1** |  |  |  |  |  |  |
| 2012 | Holt | Archives of General Psychiatry | 20 / 17 | 35 / 34 | 22945619 |  |  |  |  |  |  |  |  |  |  |  |  |  |  | **1** |  |  |  |  |  |  |
| 2012 | Morris | Translational Psychiatry | 12 / 15 | 44 / 35 | 22832855 |  |  |  |  |  |  |  |  |  |  |  |  |  |  | **1** |  |  |  |  |  |  |
| 2012 | Kim | Journal of Psychiatric Research | 16 / 17 | 30 / 28 | 22770670 |  |  |  |  |  |  |  |  |  |  |  |  |  |  | **1** |  |  |  |  |  |  |
| 2012 | Li | Schizophrenia Research | 12 / 12 | 30 / 29 | 22113155 |  |  |  |  |  |  |  |  |  |  |  |  |  |  | **1** |  |  |  |  |  |  |
| 2011 | Lakis | Psychiatry Research: Neuroimaging | 37 / 37 | 32 / 32 | 22079660 |  |  |  |  |  |  |  |  |  |  |  |  |  |  | **1** | **1** |  |  |  |  |  |
| 2012 | de la Fuente-Sandoval | Journal of Psychiatric Research | 12 / 13 | 24 / 26 | 21955439 |  |  |  |  |  |  |  |  |  |  |  |  |  |  | **1** |  |  |  |  |  |  |
| 2011 | Leitman | Biological Psychiatry | 23 / 28 | 34 / 34 | 21762876 |  |  |  |  |  |  |  |  |  |  |  |  |  |  | **1** | **1** |  |  |  |  |  |
| 2011 | Lepage | Psychological Medicine | 26 / 26 | 32 / 28 | 21284912 |  |  |  |  |  |  |  |  |  |  |  |  |  |  | **1** |  |  |  |  |  |  |
| 2011 | Pinkham | American Journal of Psychiatry | 35 / 37 | 36 / 36 | 21159729 |  |  |  |  |  |  |  |  |  |  |  |  |  |  | **1** |  |  |  |  |  |  |
| 2010 | Habel | Schizophrenia Research | 17 / 17 | 34 / 34 | 20663646 |  |  |  |  |  |  |  |  |  |  |  |  |  |  | **1** |  |  |  |  |  |  |
| 2010 | de la Fuente-Sandoval | Psychiatry Research: Neuroimaging | 12 / 13 | 24 / 26 | 20609569 |  |  |  |  |  |  |  |  |  |  |  |  |  |  | **1** |  |  |  |  |  |  |
| 2010 | Rauch | Psychiatry Research: Neuroimaging | 12 / 12 | 28 / 27 | 20488680 |  |  |  |  |  |  |  |  |  |  |  |  |  |  | **1** |  |  |  |  |  |  |
| 2010 | Harvey | Journal of Psychiatric Research | 30 / 26 | 30 / 31 | 20116072 |  |  |  |  |  |  |  |  |  |  |  |  |  |  | **1** | **1** |  |  |  |  |  |
| 2010 | Mier | Psychological Medicine | 16 / 16 | 34 / 37 | 20056024 |  |  |  |  |  |  |  |  |  |  |  |  |  |  | **1** | **1** |  |  |  |  |  |
| 2009 | Blasi | Psychiatry Research: Neuroimaging | 12 / 12 | 28 / 27 | 19428222 |  |  |  |  |  |  |  |  |  |  |  |  |  |  | **1** |  |  |  |  |  |  |
| 2009 | Park | Neuroscience Letters | 15 / 16 | NR / NR | 19409961 |  |  |  |  |  |  |  |  |  |  |  |  |  |  | **1** |  |  |  |  |  |  |
| 2009 | Kumari | Schizophrenia Research | 26 / 14 | 34 / 33 | 19230621 |  |  |  |  |  |  |  |  |  |  |  |  |  |  | **1** |  |  |  |  |  |  |
| 2009 | Reske | Journal of Psychiatric Research | 18 / 18 | 32 / 32 | 19056093 |  |  |  |  |  |  |  |  |  |  |  |  |  |  | **1** | **1** |  |  |  |  |  |
| 2009 | Seiferth | Neuropsychopharmacology | 12 / 12 | 18 / 18 | 18580874 |  |  |  |  |  |  |  |  |  |  |  |  |  |  | **1** | **1** |  |  |  |  |  |
| 2008 | Michalopoulou | British Journal of Psychiatry | 11 / 9 | 35 / 32 | 18310578 |  |  |  |  |  |  |  |  |  |  |  |  |  |  | **1** |  |  |  |  |  |  |
| 2008 | Hall | Biological Psychiatry | 19 / 24 | 38 / 35 | 18295746 |  |  |  |  |  |  |  |  |  |  |  |  |  |  | **1** |  |  |  |  |  |  |
| 2008 | Fakra | Schizophrenia Research | 14 / 14 | 37 / 35 | 18234477 |  |  |  |  |  |  |  |  |  |  |  |  |  |  | **1** |  |  |  |  |  |  |
| 2007 | Gur | Archives of General Psychiatry | 16 / 17 | 30 / 25 | 18056543 |  |  |  |  |  |  |  |  |  |  |  |  |  |  | **1** |  |  |  |  |  |  |
| 2007 | Reske | Journal of Psychiatric Research | 10 / 10 | 37 / 35 | 17467008 |  |  |  |  |  |  |  |  |  |  |  |  |  |  | **1** |  |  |  |  |  |  |
| 2007 | Taylor | Biological Psychiatry | 23 / 15 | 39 / 39 | 17434455 |  |  |  |  |  |  |  |  |  |  |  |  |  |  | **1** |  |  |  |  |  |  |
| 2007 | Williams | Psychiatry Research: Neuroimaging | 27 / 13 | 27 / 25 | 17398080 |  |  |  |  |  |  |  |  |  |  |  |  |  |  | **1** |  |  |  |  |  |  |
| 2007 | Russell | Neuropsychologia | 15 / 10 | 45 / 36 | 16814818 |  |  |  |  |  |  |  |  |  |  |  |  |  |  | **1** |  |  |  |  |  |  |
| 2006 | Surguladze | Biological Psychiatry | 15 / 11 | 43 / 39 | 16487943 |  |  |  |  |  |  |  |  |  |  |  |  |  |  | **1** |  |  |  |  |  |  |
| 2006 | Holt | Schizophrenia Research | 15 / 16 | 48 / 48 | 16377154 |  |  |  |  |  |  |  |  |  |  |  |  |  |  | **1** |  |  |  |  |  |  |
| 2005 | Johnston | European Journal of Neuroscience | 11 / 15 | 34 / 30 | 16176365 |  |  |  |  |  |  |  |  |  |  |  |  |  |  | **1** |  |  |  |  |  |  |
| 2005 | Holt | Biological Psychiatry | 18 / 16 | 45 / 44 | 15860342 |  |  |  |  |  |  |  |  |  |  |  |  |  |  | **1** |  |  |  |  |  |  |
| 2004 | Takahashi | Neuroimage | 15 / 15 | 29 / 29 | 15219596 |  |  |  |  |  |  |  |  |  |  |  |  |  |  | **1** |  |  |  |  |  |  |
| 2004 | Williams | American Journal of Psychiatry | 27 / 22 | 27 / 27 | 14992974 |  |  |  |  |  |  |  |  |  |  |  |  |  |  | **1** |  |  |  |  |  |  |
| 2004 | Mitchell | British Journal of Psychiatry | 12 / 13 | 46 / 32 | 14990520 |  |  |  |  |  |  |  |  |  |  |  |  |  |  | **1** |  |  |  |  |  |  |
| 2003 | Paradiso | American Journal of Psychiatry | 18 / 17 | 30 / 30 | 14514490 |  |  |  |  |  |  |  |  |  |  |  |  |  |  | **1** |  |  |  |  |  |  |
| 2002 | Gur | American Journal of Psychiatry | 14 / 14 | 29 / 27 | 12450947 |  |  |  |  |  |  |  |  |  |  |  |  |  |  | **1** |  |  |  |  |  |  |
| 2002 | Taylor | Schizophrenia Research | 14 / 13 | 36 / 30 | 12409155 |  |  |  |  |  |  |  |  |  |  |  |  |  |  | **1** |  |  |  |  |  |  |
| 2002 | Kosaka | Schizophrenia Research | 12 / 12 | 26 / 24 | 12165379 |  |  |  |  |  |  |  |  |  |  |  |  |  |  | **1** |  |  |  |  |  |  |
| 2012 | Pedersen | Schizophrenia Research | 15 / 14 | 29 / 30 | 22406281 |  |  |  |  |  |  |  |  |  |  |  |  |  |  |  | **1** |  |  |  |  |  |
| 2012 | Gizewski | Addiction Biology | 12 / 12 | 38 / 37 | 22340281 |  |  |  |  |  |  |  |  |  |  |  |  |  |  |  | **1** |  |  |  |  |  |
| 2012 | Derntl | Schizophrenia Research | 15 / 15 | 34 / 30 | 22306196 |  |  |  |  |  |  |  |  |  |  |  |  |  |  |  | **1** |  |  |  |  |  |
| 2011 | Lee | Social Neuroscience | 12 / 13 | 38 / 43 | 22050432 |  |  |  |  |  |  |  |  |  |  |  |  |  |  |  | **1** |  |  |  |  |  |
| 2012 | Das | Schizophrenia Research | 20 / 19 | 35 / 34 | 21943555 |  |  |  |  |  |  |  |  |  |  |  |  |  |  |  | **1** |  |  |  |  |  |
| 2011 | Brune | Neuroimage | 22 / 26 | 27 / 29 | 21147235 |  |  |  |  |  |  |  |  |  |  |  |  |  |  |  | **1** |  |  |  |  |  |
| 2011 | Park | Acta Psychiatrica Scandinavica | 14 / 15 | 30 / 28 | 20712827 |  |  |  |  |  |  |  |  |  |  |  |  |  |  |  | **1** |  |  |  |  |  |
| 2010 | Lee | Psychiatry Research: Neuroimaging | 15 / 18 | 26 / 26 | 20080395 |  |  |  |  |  |  |  |  |  |  |  |  |  |  |  | **1** |  |  |  |  |  |
| 2009 | Benedetti | Schizophrenia Research | 24 / 20 | 37 / 35 | 19632816 |  |  |  |  |  |  |  |  |  |  |  |  |  |  |  | **1** |  |  |  |  |  |
| 2009 | Walter | Social Cognitive and Affective Neuroscience | 12 / 12 | 30 / 25 | 19287044 |  |  |  |  |  |  |  |  |  |  |  |  |  |  |  | **1** |  |  |  |  |  |
| 2008 | Andreasen | Schizophrenia Bulletin | 18 / 13 | 33 / 27 | 18559406 |  |  |  |  |  |  |  |  |  |  |  |  |  |  |  | **1** |  |  |  |  |  |
| 2008 | Brune | Neuropsychologia | 9 / 13 | 28 / 26 | 18329671 |  |  |  |  |  |  |  |  |  |  |  |  |  |  |  | **1** |  |  |  |  |  |
| 2003 | Brunet | Neuropsychologia | 7 / 8 | 31 / 23 | 12887982 |  |  |  |  |  |  |  |  |  |  |  |  |  |  |  | **1** |  |  |  |  |  |
| 2000 | Russell | American Journal of Psychiatry | 5 / 7 | 36 / 40 | 11097974 |  |  |  |  |  |  |  |  |  |  |  |  |  |  |  | **1** |  |  |  |  |  |
| 2012 | van der Meer | Schizophrenia Bulletin | 47 / 21 | 34 / 30 | 23104865 |  |  |  |  |  |  |  |  |  |  |  |  |  |  |  |  | **1** |  |  |  |  |
| 2012 | Kambeitz-Ilankovic | Schizophrenia Bulletin | 20 / 20 | 26 / 26 | 22987297 |  |  |  |  |  |  |  |  |  |  |  |  |  |  |  |  | **1** |  |  |  |  |
| 2012 | Bedford | BMC Psychiatry | 11 / 8 | 39 / 31 | 22876974 |  |  |  |  |  |  |  |  |  |  |  |  |  |  |  |  | **1** |  |  |  |  |
| 2012 | Shad | Schizophrenia Research | 17 / 15 | 40 / 44 | 22480958 |  |  |  |  |  |  |  |  |  |  |  |  |  |  |  |  | **1** |  |  |  |  |
| 2011 | Holt | Biological Psychiatry | 19 / 20 | 36 / 40 | 21144498 |  |  |  |  |  |  |  |  |  |  |  |  |  |  |  |  | **1** |  |  |  |  |
| 2010 | Kumari | Frontiers in Behavioral Neuroscience | 38 / 20 | 38 / 34 | 20179788 |  |  |  |  |  |  |  |  |  |  |  |  |  |  |  |  | **1** |  |  |  |  |
| 2010 | Murphy | Schizophrenia Research | 7 / 10 | 27 / 30 | 20051318 |  |  |  |  |  |  |  |  |  |  |  |  |  |  |  |  | **1** |  |  |  |  |
| 2010 | Kumari | Schizophrenia Bulletin | 63 / 20 | 38 / 34 | 18997158 |  |  |  |  |  |  |  |  |  |  |  |  |  |  |  |  | **1** |  |  |  |  |
| 2008 | Schnell | Brain | 15 / 15 | 30 / 31 | 18713781 |  |  |  |  |  |  |  |  |  |  |  |  |  |  |  |  | **1** |  |  |  |  |
| 2008 | Vinogradov | Cerebral Cortex | 8 / 8 | 38 / 28 | 18321870 |  |  |  |  |  |  |  |  |  |  |  |  |  |  |  |  | **1** |  |  |  |  |
| 2007 | Allen | British Journal of Psychiatry | 20 / 11 | 35 / 29 | 17267934 |  |  |  |  |  |  |  |  |  |  |  |  |  |  |  |  | **1** |  |  |  |  |
| 2006 | Ragland | Schizophrenia Research | 13 / 13 | 35 / 31 | 16814525 |  |  |  |  |  |  |  |  |  |  |  |  |  |  |  |  | **1** |  |  |  |  |
| 2004 | Blackwood | Psychological Medicine | 8 / 8 | 38 / 36 | 15099414 |  |  |  |  |  |  |  |  |  |  |  |  |  |  |  |  | **1** |  |  |  |  |
| 2013 | Bjorkquist | Psychiatry Research: Neuroimaging | 14 / 14 | 32 / 31 | 23642469 |  |  |  |  |  |  |  |  |  |  |  |  |  |  |  |  |  | **1** |  |  |  |
| 2012 | Gradin | PLoS One | 13 / 16 | 41 / 41 | 22916139 |  |  |  |  |  |  |  |  |  |  |  |  |  |  |  |  |  | **1** |  |  |  |
| 2013 | Ebisch | Social Cognitive and Affective Neuroscience | 24 / 22 | 27 / 28 | 22275166 |  |  |  |  |  |  |  |  |  |  |  |  |  |  |  |  |  | **1** |  |  |  |
| 2011 | Taylor | Journal of Psychiatric Research | 21 / 21 | 41 / 40 | 20797730 |  |  |  |  |  |  |  |  |  |  |  |  |  |  |  |  |  | **1** |  |  |  |
| 2008 | Kohler | Schizophrenia Research | 13 / 12 | 35 / 30 | 18248794 |  |  |  |  |  |  |  |  |  |  |  |  |  |  |  |  |  | **1** |  |  |  |
| 2013 | Gradin | Psychiatry Research: Neuroimaging | 14 / 18 | 43 / 40 | 23146249 |  |  |  |  |  |  |  |  |  |  |  |  |  |  |  |  |  |  | **1** |  |  |
| 2012 | Nielsen | Biological Psychiatry | 31 / 31 | 26 / 26 | 22418013 |  |  |  |  |  |  |  |  |  |  |  |  |  |  |  |  |  |  | **1** |  |  |
| 2012 | Grimm | Schizophrenia Research | 23 / 23 | 30 / 29 | 22209236 |  |  |  |  |  |  |  |  |  |  |  |  |  |  |  |  |  |  | **1** |  |  |
| 2011 | Gradin | Brain | 14 / 17 | 43 / 41 | 21482548 |  |  |  |  |  |  |  |  |  |  |  |  |  |  |  |  |  |  | **1** |  |  |
| 2010 | Waltz | Neuropsychopharmacology | 17 / 17 | 38 / 38 | 20720534 |  |  |  |  |  |  |  |  |  |  |  |  |  |  |  |  |  |  | **1** |  |  |
| 2009 | Walter | Psychopharmacology | 16 / 16 | 38 / 33 | 19521678 |  |  |  |  |  |  |  |  |  |  |  |  |  |  |  |  |  |  | **1** |  |  |
| 2009 | Schlagenhauf | Biological Psychiatry | 15 / 15 | 30 / 30 | 19195646 |  |  |  |  |  |  |  |  |  |  |  |  |  |  |  |  |  |  | **1** |  |  |
| 2006 | Juckel | Neuroimage | 10 / 10 | 27 / 32 | 16139525 |  |  |  |  |  |  |  |  |  |  |  |  |  |  |  |  |  |  | **1** |  |  |
| 2006 | Juckel | Psychopharmacology | 20 / 10 | 35 / 31 | 16721614 |  |  |  |  |  |  |  |  |  |  |  |  |  |  |  |  |  |  | **1** |  |  |
| 2006 | Postma | Psychopharmacology | 5 / 6 | 31 / 32 | 16456657 |  |  |  |  |  |  |  |  |  |  |  |  |  |  |  |  |  |  |  | **1** |  |
| 2003 | Kumari | Psychiatry Research: Neuroimaging | 7 / 6 | 40 / 33 | 12714174 |  |  |  |  |  |  |  |  |  |  |  |  |  |  |  |  |  |  |  | **1** |  |
| 2011 | Koch | British Journal of Psychiatry | 19 / 20 | 35 / 30 | 21357881 |  |  |  |  |  |  |  |  |  |  |  |  |  |  |  |  |  |  |  |  | **1** |
| 2002 | Paulus | Biological Psychiatry | 15 / 15 | 42 / 41 | 12062884 |  |  |  |  |  |  |  |  |  |  |  |  |  |  |  |  |  |  |  |  | **1** |
|  |  |  |  |  |  |  |  |  |  |  |  |  |  |  |  |  |  |  |  |  |  |  |  |  |  |  |
|  | ^= mean or if not reported, median. | | | | | | | | | | | | | | | | |  |  |  |  |  |  |  |  |  |
|  | *= includes Stroop task and other task-switching paradigms | | | | | | | | | | | | | | | | |  |  |  |  |  |  |  |  |  |
|  | **= includes smooth pursuit eye movements | | | | | | | | | | | | | | | | |  |  |  |  |  |  |  |  |  |
|  | ***= includes tasks only looking at empathy | | | | | | | | | | | | | | | | |  |  |  |  |  |  |  |  |  |
|  | NR= not reported | | | | | | | | | | | | | | | | |  |  |  |  |  |  |  |  |  |
| Note that the 3 classes in red (Reward, Decision under uncertainty, and PPI) are not included in the Brainmap taxonomy. These classes were included in the present study because they have been widely used in the schizophrenia literature. | | | | | | | | | | | | | | | | | | | | | | |  |  |  |  |
| Some studies might be classified in different groups. However, their contrasts were included in only one class. For example, a study might have used a working memory task with emotional cues, but their contrasts would specifically look at the effect of working memory or emotion. | | | | | | | | | | | | | | | | | | | | | | |  |  |  |  |
|  |  |  |  |  |  |  |  |  |  |  |  |  |  |  |  |  |  |  |  |  |  |  |  |  |  |  |
|  |  |  |  |  |  |  |  |  |  |  |  |  |  |  |  |  |  |  |  |  |  |  |  |  |  |  |
|  |  |  |  |  |  |  |  |  |  |  |  |  |  |  |  |  |  |  |  |  |  |  |  |  |  |  |
|  |  |  |  |  |  |  |  |  |  |  |  |  |  |  |  |  |  |  |  |  |  |  |  |  |  |  |
|  |  |  |  |  |  |  |  |  |  |  |  |  |  |  |  |  |  |  |  |  |  |  |  |  |  |  |
|  |  |  |  |  |  |  |  |  |  |  |  |  |  |  |  |  |  |  |  |  |  |  |  |  |  |  |
|  |  |  |  |  |  |  |  |  |  |  |  |  |  |  |  |  |  |  |  |  |  |  |  |  |  |  |
|  |  |  |  |  |  |  |  |  |  |  |  |  |  |  |  |  |  |  |  |  |  |  |  |  |  |  |

***References***

**1.** Watts DJ, Strogatz SH. Collective dynamics of 'small-world' networks. *Nature* 1998;393(6684):440-442.

**2.** Newman ME. Modularity and community structure in networks. *Proc Natl Acad Sci U S A* Jun 6 2006;103(23):8577-8582.

**3.** Guimera R, Nunes Amaral LA. Functional cartography of complex metabolic networks. *Nature* Feb 24 2005;433(7028):895-900.

**4.** Opsahl T, Colizza V, Panzarasa P, Ramasco JJ. Prominence and control: the weighted rich-club effect. *Physical Review Letters* Oct 17 2008;101(16):168702.

**5.** Crossley NA, Mechelli A, Vertes PE, Winton-Brown TT, Patel AX, Ginestet CE, McGuire P, Bullmore ET. Cognitive relevance of the community structure of the human brain functional coactivation network. *P Natl Acad Sci USA* Jul 9 2013;110(28):11583-11588.

**6.** Goldman-Rakic PS, Selemon LD. Functional and anatomical aspects of prefrontal pathology in schizophrenia. *Schizophrenia Bulletin* 1997;23(3):437-458.
